# Supplementary material for: Rational engineering of a thermostable α-oxoamine synthase biocatalyst expands the substrate scope and synthetic applicability
Source: Commun Chem. 2025 Mar 13;8:78. doi: 10.1038/s42004-025-01448-8 (PMC11906848; doi:10.1038/s42004-025-01448-8)
Supplement: Supplementary file 2 — Supplementary Information [file 42004_2025_1448_MOESM2_ESM.pdf]

Supporting Information for

Rational Engineering of a Thermostable α-Oxoamine Synthase Biocatalyst Expands the Substrate Scope and Synthetic Applicability

Ben Ashley,[a] Sam Mathew,[a] Mariyah Sajjad,[a] Yaoyi Zhu,[a] Nikita Novikovs,[a] Arnaud Baslé,[b] Jon Marles-Wright,[b] and Dominic J. Campopiano[a]*

[a]School of Chemistry, University of Edinburgh, David Brewster Road EH9 3FJ, Edinburgh, UK
[b]School of Natural and Evironmental Sciences, Newcastle University, Devonshire Building NE1 7RU, Newcastle-Upon-Tyne, UK
*Dominic.Campopiano@ed.ac.uk

Contents

Contents 1

Sequences & Mutagenesis 3

HPLC Methods 4

Figures S1-S23 5

Figure S1. UV-vis absorption profiles of wild-type ThAOS during titration against various amino-acids (0-16 mM). 5

Figure S2. UV-vis absorption profile of ThAOS during reactions with different l-amino-acids and acetyl-CoA. 6

Figure S3. Optimisation of the heat purification of ThAOS. 7

Figure S4. Chemical structures of the unnatural amino-acids (UAAs) used in this study. 8

Figure S5. Kinetic characterisation of the mutants against several of the reported substrates. 9

Figure S6. Summary of the performance of the ThAOS V79 variants 10

Figure S7. UV-visible analysis to investigate quinonoid formation of the ThAOS V79G variant. 11

Figure S8. Thermostability of ThAOS wild type and variants at 90 °C and 70 °C (after two hours incubation). 12

Figure S9A. Proton NMR of acetyl-SNAC. 13

Figure S9B. Carbon NMR of acetyl-SNAC. 13

Figure S10. Activity screen of ThAOS wild type and three mutants (V79A, V79G and V79S) using three amino acids and the acetyl-SNAc thioester substrate 14

Figure S11. Pyrrole 1 synthesis using V79A using different concentrations of acetyl-SNAc. 15

Figure S12A. Analysis of isolated pyrrole (1). 16

Figure S12B. Analysis of isolated pyrrole (1). 17

Figure S13. Analysis of isolated pyrrole (1). 18
Figure S14. Mass spectrometry analysis of the ThAOS V79A variant incubated with Gly and acetyl-CoA. 19
Figure S15. Formation of pyrrole from l-Alanine. 20
Figure S16. Formation of pyrrole from l-Allyglycine. 21
Figure S17. Formation of pyrrole from l-Cyclopropylglycine. 22
Figure S18. UV-vis analysis of L-Pen binding to ThAOS. 23
Figure S19B. Side-chain: L-Pen ligand interactions. 24
Figure S20. Structural alignment of ThAOS V79A and homologous cysteine desulfurase (CSD) enzymes (CSDs). 26
Figure S21. Binding of L-Pen in ThAOS V79A and cysteine desulfurases (CSDs). 27
Figure S22. Multiple sequence alignment of ThAOS V79A variant and cysteine desulfurase (CysD) enzymes. 28
Figure S23. Multiple sequence and structural alignment of various AOS biocatalysts. 29
Tables S1-S6 30
Table S1. Primer sequences used to generate all mutants used in this study. 30
Table S2. Full kinetic characterisation of ThAOS V79A and ThAOS V79G with various amino-acid substrates. 31
Table S3. Summary of saturation mutagenesis at ThAOS V79. 32
Table S4. Heat map illustrating the full Claisen-condensation substrate range of ThAOS V79G. 33
Table S5. Heat-map of the activity of ThAOS V79A with a panel of L-amino-acid/acyl-CoA substrate pairings. 34
Table S6. Crystallographic data collection and refinement statistics. 36

Sequences & Mutagenesis

Mutant DNA was prepared according to the overlapping primerp site-directed mutagenesis method as according to Liu *et al*.[1] Saturation mutagenesis was initially attempted using the 22c trick,[2] and mutants which were not obtained in the initial screen were prepared individually or in small groups. Primers were purchased from Merck Life Sciences and sequences are outlined in Table S1. The *Th*AOS DNA gene sequence is as below. The recombinant *Th*AOS protein sequence with V79 highlighted in red. Uniprot code: Q5SHZ8.

Gene sequence:
ATCGATCTCGATCCCGCGAAATTAATACGACTCACTATAGGGGAATTGTGAGCGGATAACAATTCCCCTCTAG
AAATAATTTTGTTTAACTTTAAGAAGGAGATATACATATGTCGTACTACCATCACCATCACCATCACGATTACG
ACATCCCAACGACCGAAAACCTGTATTTTCAGGGCGCCATGGGCAGCCTGGATCTGCGTGCGCGTGTGCGTG
AAGAACTGGAGCGTCTGAAGCGTGAAGGTCTGTATATTAGCCCGAAAGTGCTGGAAGCGCCGCAGGAACCG
GTGACCCGTGTTGAAGGCCGTGAGGTGGTTAACCTGGCGAGCAACAACTACCTGGGTTTTGCGAACCACCCG
TATCTGAAGGAAAAAGCGCGTCAATACCTGGAGAAATGGGGTGCGGGTAGCGGTGCGGTGCGTACCATCGC
GGGCACCTTCACCTATCACGTTGAACTGGAGGAAGCGCTGGCGCGTTTTAAAGGTACCGAGAGCGCGCTGGT
GCTGCAGAGCGGTTTCACCGCGAACCAAGGCGTTCTGGGTGCGCTGCTGAAGGAAGGCGACGTGGTTTTTAG
CGATGAGCTGAACCACGCGAGCATCATTGACGGTCTGCGTCTGACCAAAGCGACCCGTCTGGTGTTCCGTCAC
GCGGATGTTGCGCACCTGGAGGAACTGCTGAAGGCGCACGACACCGATGGTCTGAAACTGATTGTGACCGAC
GGCGTTTTTAGCATGGACGGTGATATCGCGCCGCTGGATAAGATTGTGCCGCTGGCGAAGAAATACAAAGCG
GTGGTTTATGTGGACGATGCGCACGGCAGCGGTGTTCTGGGCGAAAAGGGCAAAGGTACCGTGCACCACTTC
GGTTTTCACCAGGACCCGGATGTGGTTCAAGTGGCGACCCTGAGCAAAGCGTGGGCGGGTATCGGTGGCTAC
GCGGCGGGTGCGCGTGAGCTGAAGGACCTGCTGATTAACAAAGCGCGTCCGTTCCTGTTTAGCACCAGCCAC
CCGCCGGCGGTGGTTGGTGCGCTGCTGGGTGCGCTGGAACTGATCGAGAAGGAACCGGAGCGTGTGGAACG
TCTGTGGGAGAACACCCGTTATTTCAAACGTGAGCTGGCGCGTCTGGGCTACGATACCCTGGGTAGCCAGACC
CCGATCACCCCGGTTCTGTTCGGTGAAGCGCCGCTGGCGTTTGAGGCGAGCCGTCTGCTGCTGGAGGAAGGC
GTGTTCGCGGTTGGCATTGGTTTTCCGACCGTGCCGCGTGGTAAAGCGCGTATCCGTAACATTGTTACCGCGG
CGCACACCAAAGAGATGCTGGACAAGGCGCTGGAGGCGTATGAAAAGGTTGGCAAACGTCTGGGTATTATCC
GCTAACAAAGCCCGAAAGGAAGCTGAGTTGCTGCTGCCACCGCTGAGCAATAACTAGCATAACCCCTTGGGG
CCTCTAAACGGGTCTTGAGGGGTTTTTTGCTGAA

*Th*AOS Expression construct:
HHHHHHDYDIPTTENLYFQGAMGSLDLRARVREELERLKREGLYISPKVLEAPQEPVTRVEGREVVNLASNNYLGF
ANHPYLKEKARQYLEKWGAGSGA**V**RTIAGTFTYHVELEEALARFKGTESALVLQSGFTANQGVLGALLKEGDVVFS
DELNHASIIDGLRLTKATRLVFRHADVAHLEELLKAHDTDGLKLIVTDGVFSMDGDIAPLDKIVPLAKKYKAVVYVDD
AHGSGVLGEKGKGTVHHFGFHQDPDVVQVATLSKAWAGIGGYAAGARELKDLLINKARPFLFSTSHPPAVVGALL
GALELIEKEPERVERLWENTRYFKRELARLGYDTLGSQTPITPVLFGEAPLAFEASRLLLEEGVFAVGIGFPTVPRGKA
RIRNIVTAAHTKEMLDKALEAYEKVGKRLGIIR.

HPLC Methods

Method 1

Quenched reaction mix (10 µL) was injected onto a Phenomenex Luna 5 µm C18 100 Å HPLC column. Samples were eluted with 5% MeCN/95% water/0.1% TFA for 7.5 min followed by a 2.5 min gradient to 55% MeCN/0.1% TFA. This concentration was maintained for 10 min and then returned to 95% water/0.1% TFA over a 2.5 min gradient, and washed with 95% water/0.1% TFA for an additional 2.5 min. The eluent was continuously monitored at 260 nm.

Method 2

Quenched reaction mix (10 µL) was injected onto a Phenomenex Luna 5 µm C18 100 Å HPLC column. Samples were eluted with 5% MeCN/95% water/0.1% TFA for 12.5 min followed by a 2.5 min gradient to 95% MeCN/0.1% TFA. This concentration was maintained for 10 min and then returned to 95% water/0.1% TFA over a 2.5 min gradient, and washed with 95% water/0.1% TFA for an additional 2.5 min. The eluent was continuously monitored at 260 nm.

Figures S1-S23

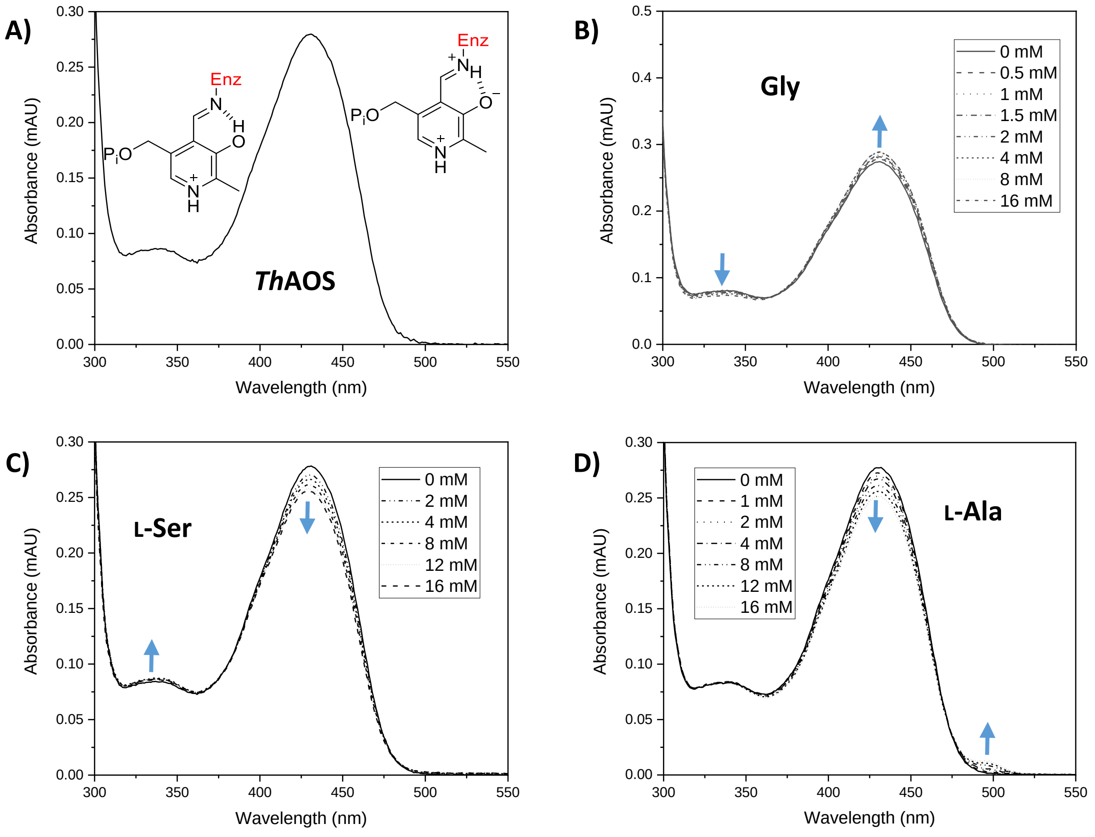

Figure S1. UV-vis absorption profiles of wild-type ThAOS during titration against various amino-acids (0-16 mM). A) UV-vis spectrum of the resting state of recombinant *Th*AOS with characteristic absorbance maxima at 330 and 425 nm (20 mM HEPES buffer pH 7.5). B) With Gly. C) With L-Ser. D) With L-Ala.

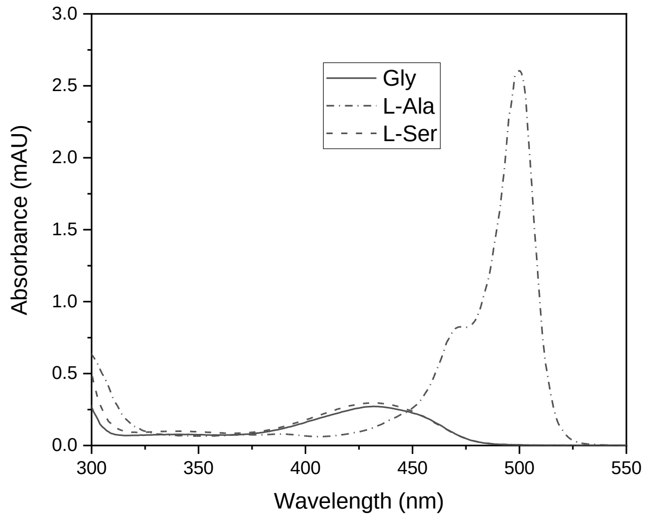

Figure S2. UV-vis absorption profile of ThAOS during reactions with different L*-amino-acids and acetyl-CoA.* Reactions were initiated by addition of acetyl-CoA (0.5 mM) to a solution containing *Th*AOS (2 mgmL-1) and amino-acid (16 mM) in HEPES buffer (pH 7.5) at room temperature, mixed and the spectrum was recorded immediately.

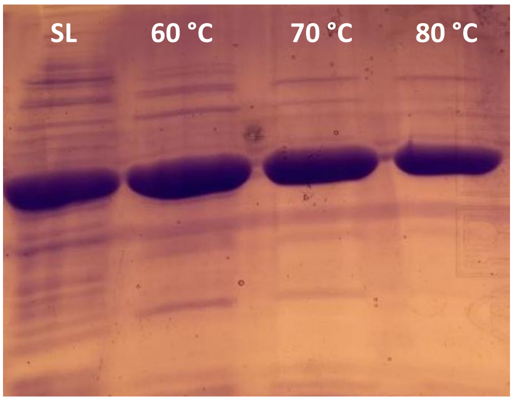

Figure S3. Optimisation of the heat purification of ThAOS. The *Th*AOS-harbouring soluble cell lysate (SL) from *E. coli* expression was incubated at the noted temperatures for 30 minutes, prior to precipitation of insoluble cell debris by centrifugation. The supernatant was analysed by SDS-PAGE.

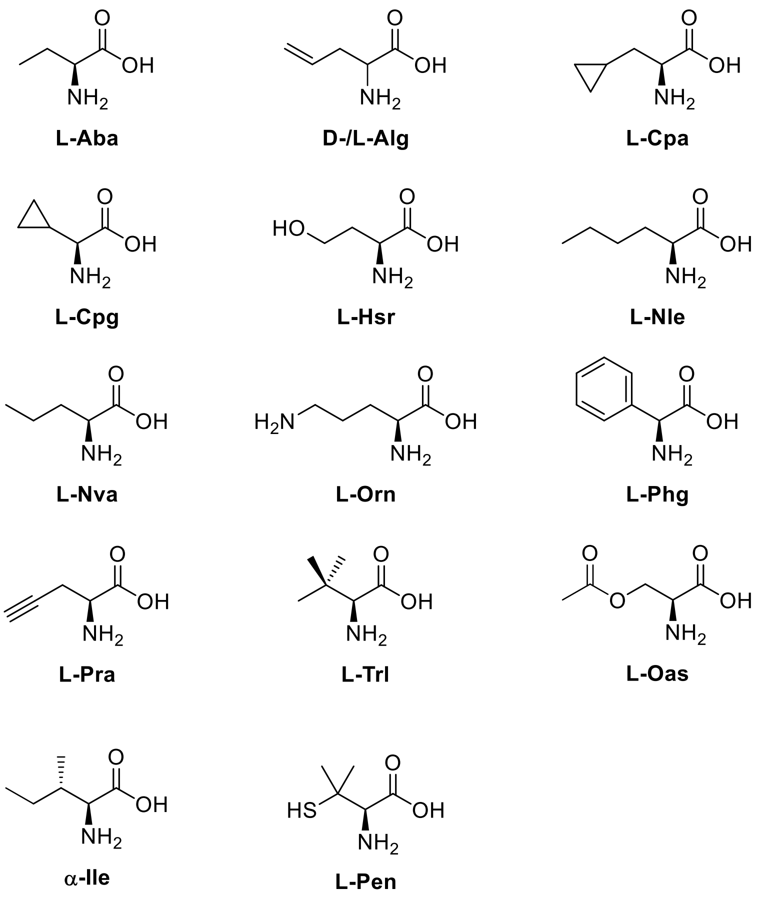

Figure S4. Chemical structures of the unnatural amino-acids (UAAs) used in this study.
The 13 substrates shown are : L-Aba, D-/L-Alg, L-Cpa, L-Cpg, L-Hsr, L-Nle, L-Nva, L-Orn, L-Phg, L-Pra, L-Trl, L-Oas and α-Ile. The structure of L-penicillamine (L-Pen), the well known PLP-dependent enzyme inhibitor is also shown.

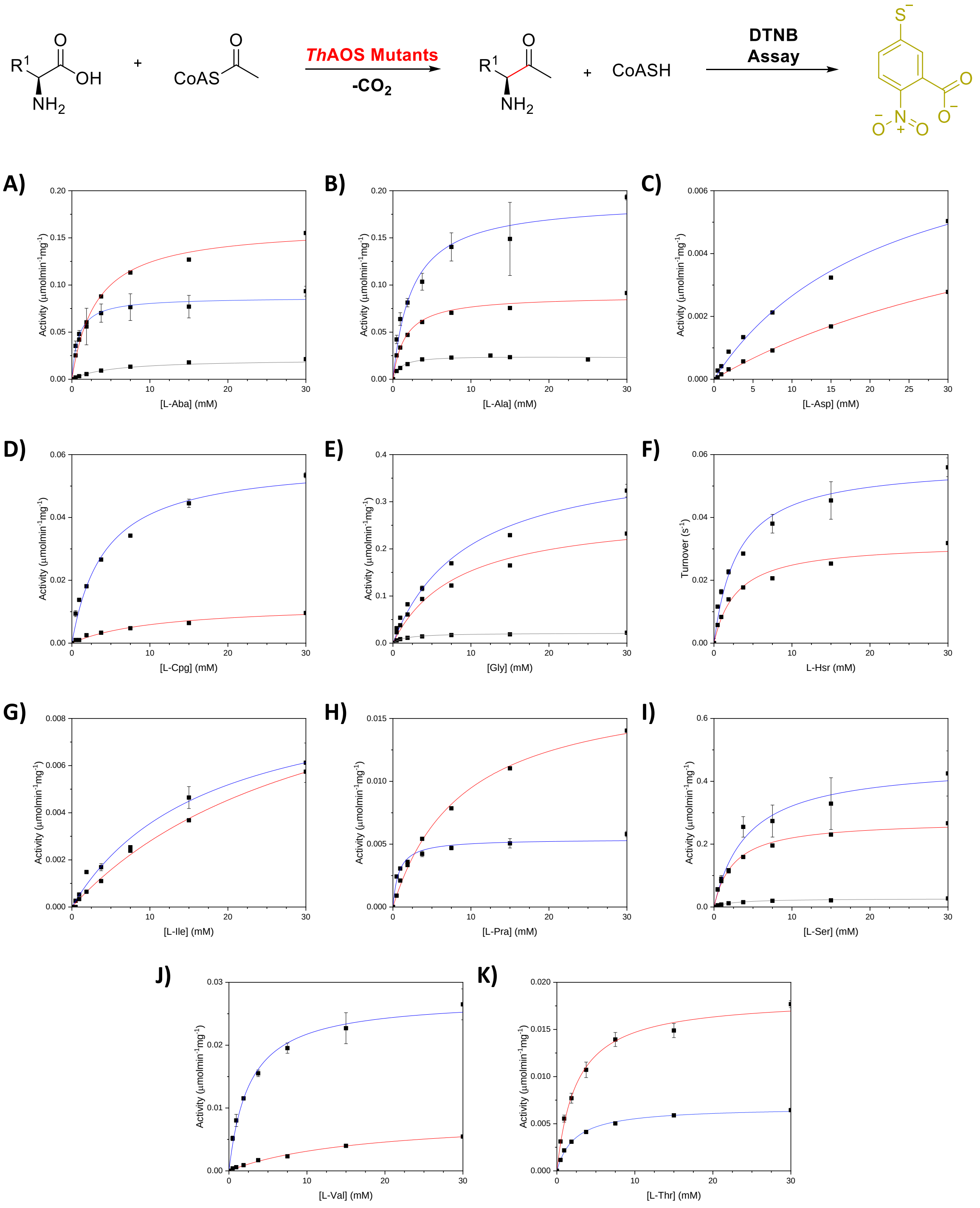

Figure S5. Kinetic characterisation of the mutants against several of the reported substrates. *Th*AOS data points are fitted in grey, *Th*AOS V79A in red and V79G in blue. Amino-acid substrates of varied concentrations were incubated with enzyme (1 mgmL-1), acetyl-CoA (1 mM) and DTNB (0.5 mM) in HEPES buffer (20 mM, pH 7.5) at 50 °C.

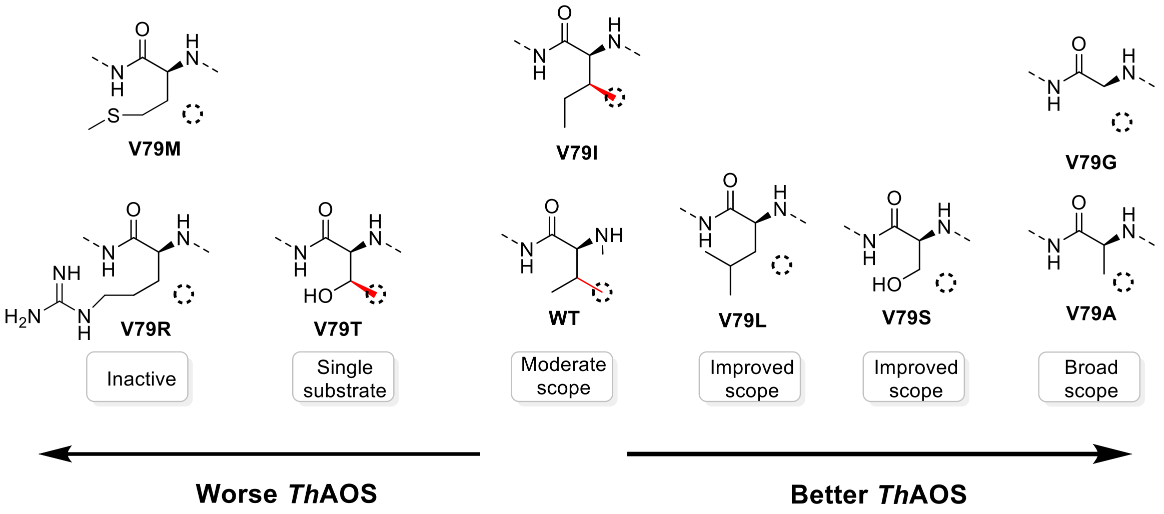

Figure S6. Summary of the performance of the ThAOS V79 variants. Structures of the amino acid sidechains of eight *Th*AOS V79 variants; six active (V79I, V79I, V79L, V79S, V79A, V79G) and two inactive (V79R, V79M). The β-methyl position of the side-chain is illustrated in red/circled.

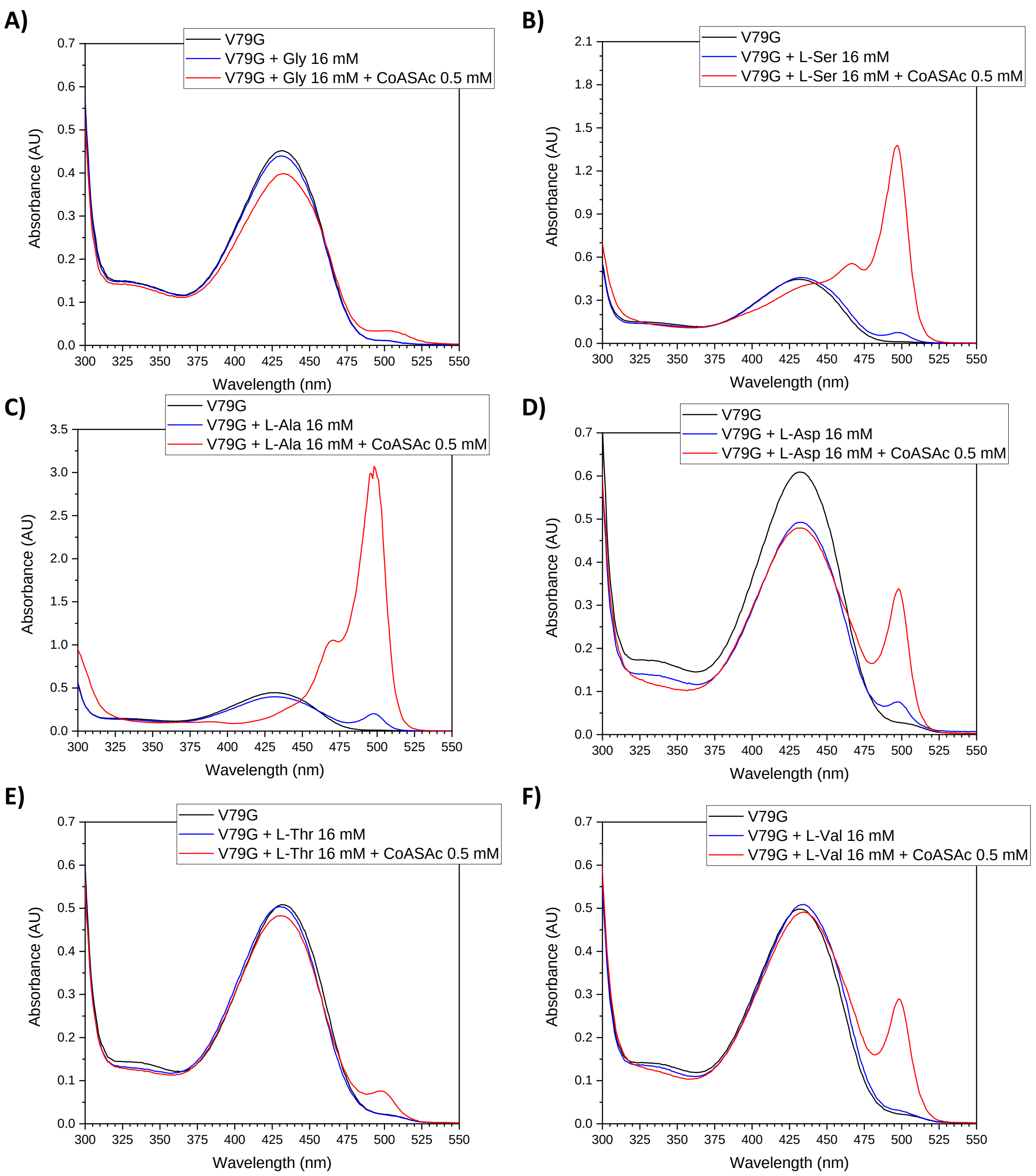

Figure S7. UV-visible analysis to investigate quinonoid formation of the ThAOS V79G variant. The UV-vis profile of the ThAOS V79G variant was measured as purified (black line), in the presence of each amino acid substrate alone (16 mM, blue line) and in the presence of acetyl-CoA (0.5 mM, red line) at room temperature. The substrates used were (A) Gly (B) L-Ser (C) L-Ala (D) L-Asp (E) L-Thr and (F) L-Val.

(A)

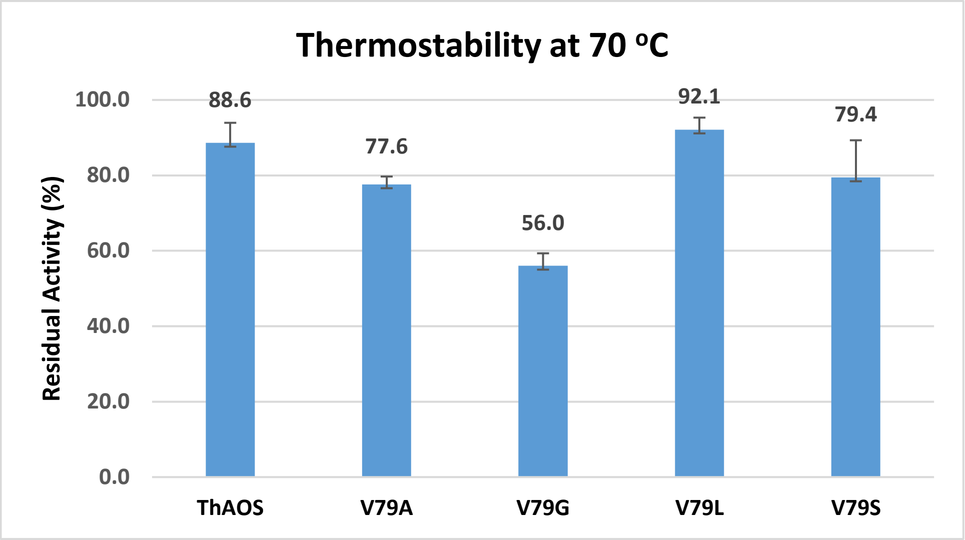

(B)

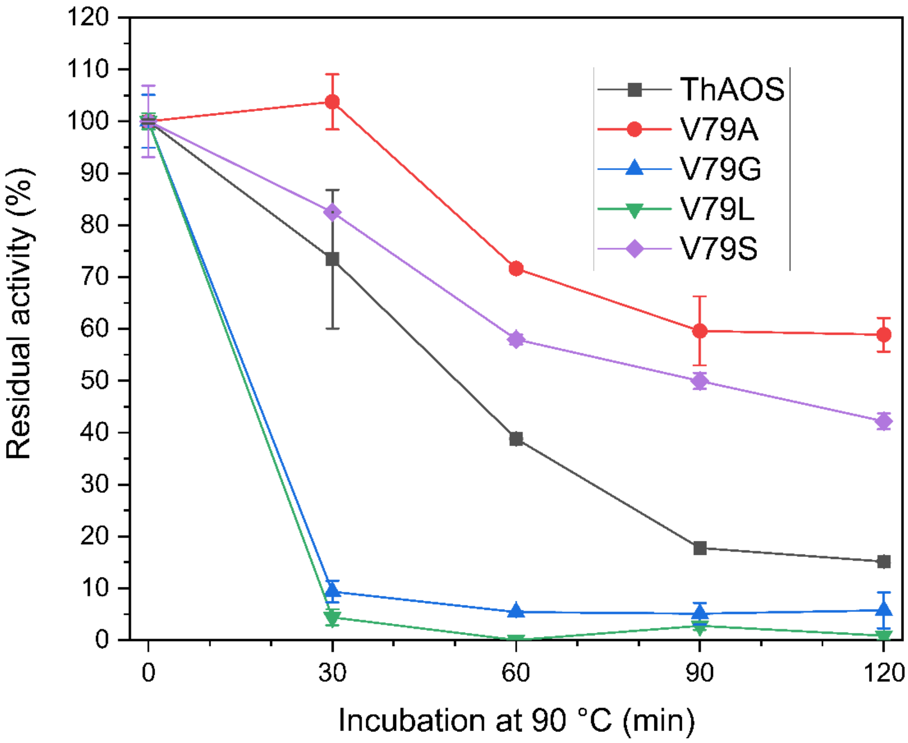

Figure S8. Thermostability of ThAOS wild type and variants at 90 °C and 70 °C (after two hours incubation). **(A)** Thermostability, defined by % residual activity remaining after incubation at 70 °C. **(B)**. Residual activity (% compare to time 0) after incubation at at 90 °C for 0-120 minutes. The reaction samples were carried out in triplicate and the measurements were taken from distinct samples. The mean values are mentioned above each bar.

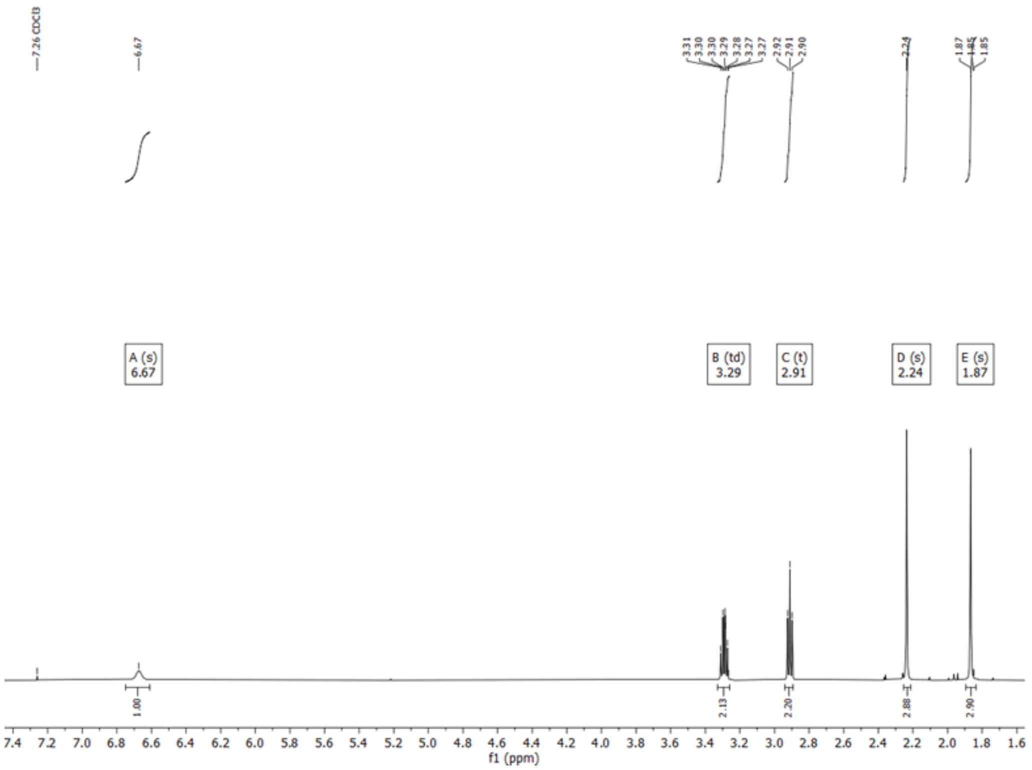

Figure S9A. Proton NMR of acetyl-SNAC. ^{1}H NMR (500 MHz, $CDCl_3$) δ 6.67 (s, 1H), 3.29 (td, J = 6.7, 5.7 Hz, 2H), 2.91 (t, J = 6.7 Hz, 2H), 2.24 (s, 3H), 1.87 (s, 3H).

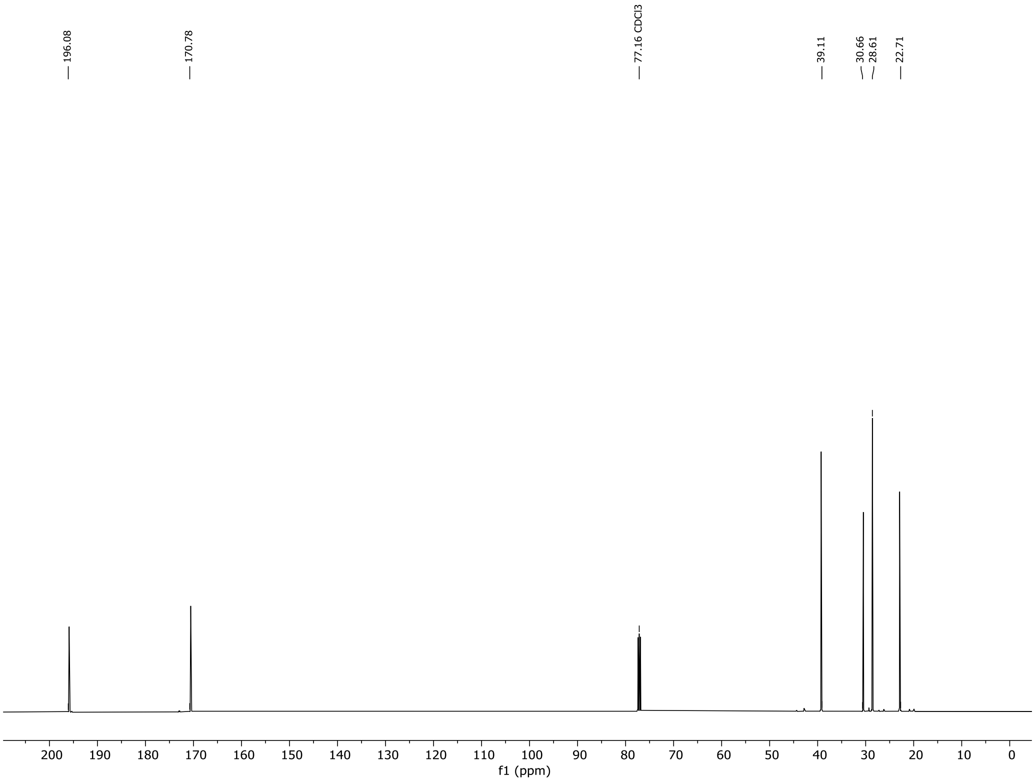

Figure S9B. Carbon NMR of acetyl-SNAC. ^{13}C NMR (125 MHz, $CDCl_3$) δ 195.9, 170.6, 39.3, 30.5, 28.6, 22.9.

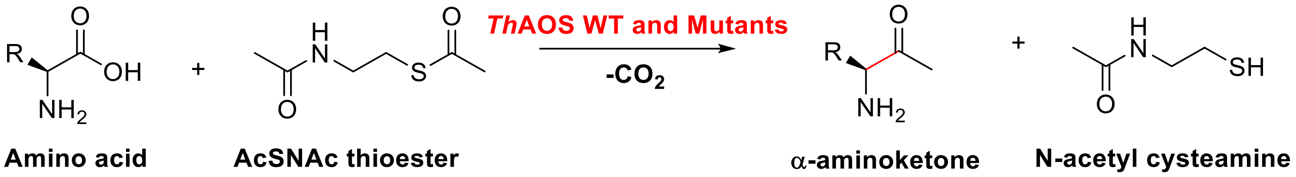

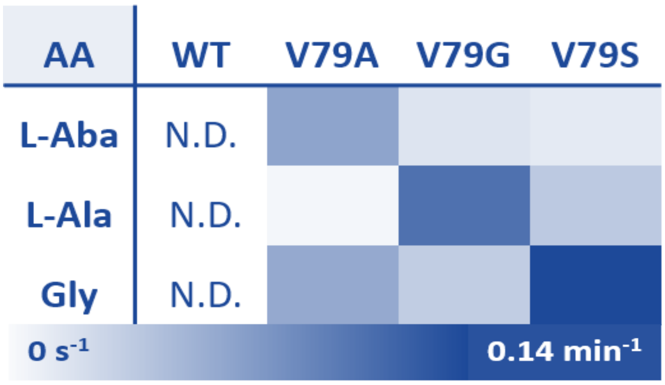

Figure S10. Activity screen of ThAOS wild type and three mutants (V79A, V79G and V79S) using three amino acids and the acetyl-SNAc thioester substrate. The *Th*AOS mutants (4 mgmL^{-1}) were incubated with amino-acid (16 mM), acetyl-SNAc (16 mM) and DTNB (0.5 mM) at 50 °C with continuous monitoring at 412 nm for 20 min. The N-acetyl cysteamine product is detected using the DTNB reagent. The scale bar and heat map describes the catalytic activity with a maximum rate of 0.14 min^{-1} for the combination of glycine and acetyl-SNAc with the *Th*AOS V79S mutant. It is worth noting that the ThAOS wild type enzyme displays no activity with the acetyl-SNAc substrate.

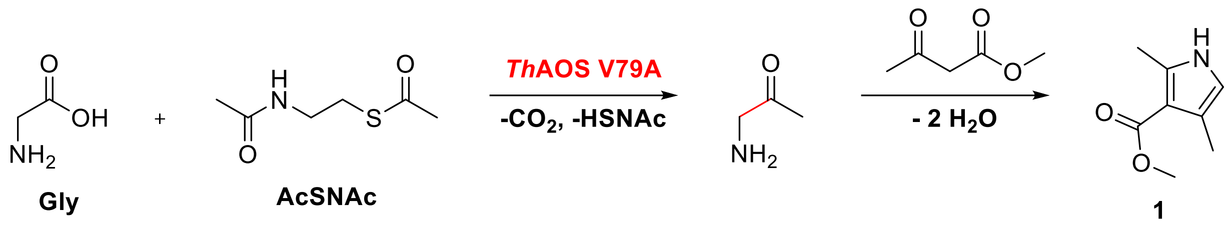

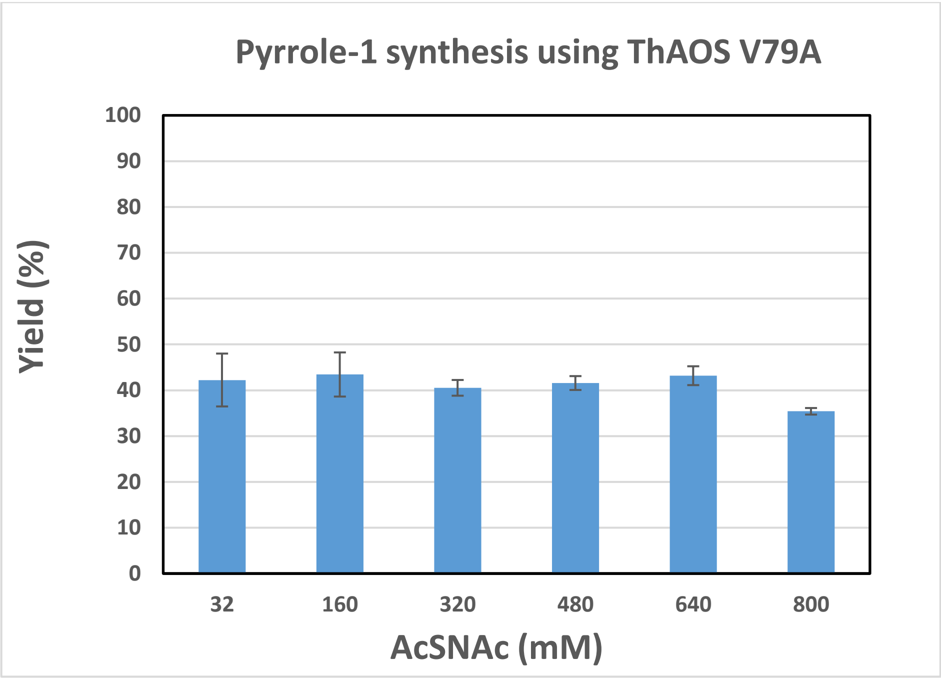

Figure S11. Pyrrole 1 synthesis using V79A using different concentrations of acetyl-SNAc. Reaction condition: 32 mM glycine, 32 mM methyl acetoacetate, 32-800 mM AcSNAc, ThAOS V79A (15 mg/mL), 100 mM HEPES buffer (pH 7.5) at 60 oC for 24 hrs. The reaction samples were carried out in triplicate and the measurements were taken from distinct samples. The mean values are mentioned above each bar.

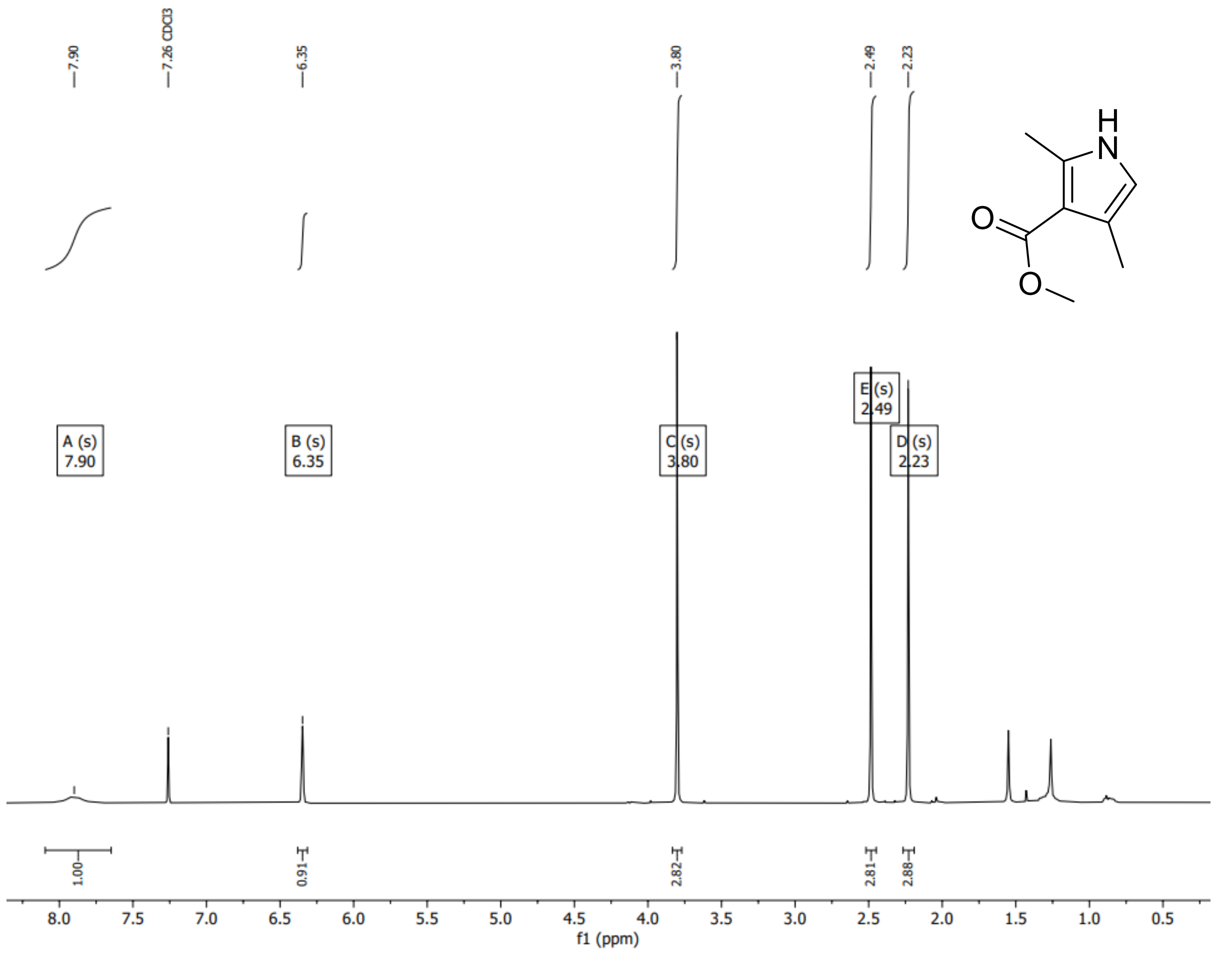

Figure S12A. Analysis of isolated pyrrole (1). 1H NMR (400 MHz, CDCl3) δ 7.90 (s, 1H), 6.35 (s, 1H), 3.80 (s, 3H), 2.49 (s, 3H), 2.23 (s, 3H).

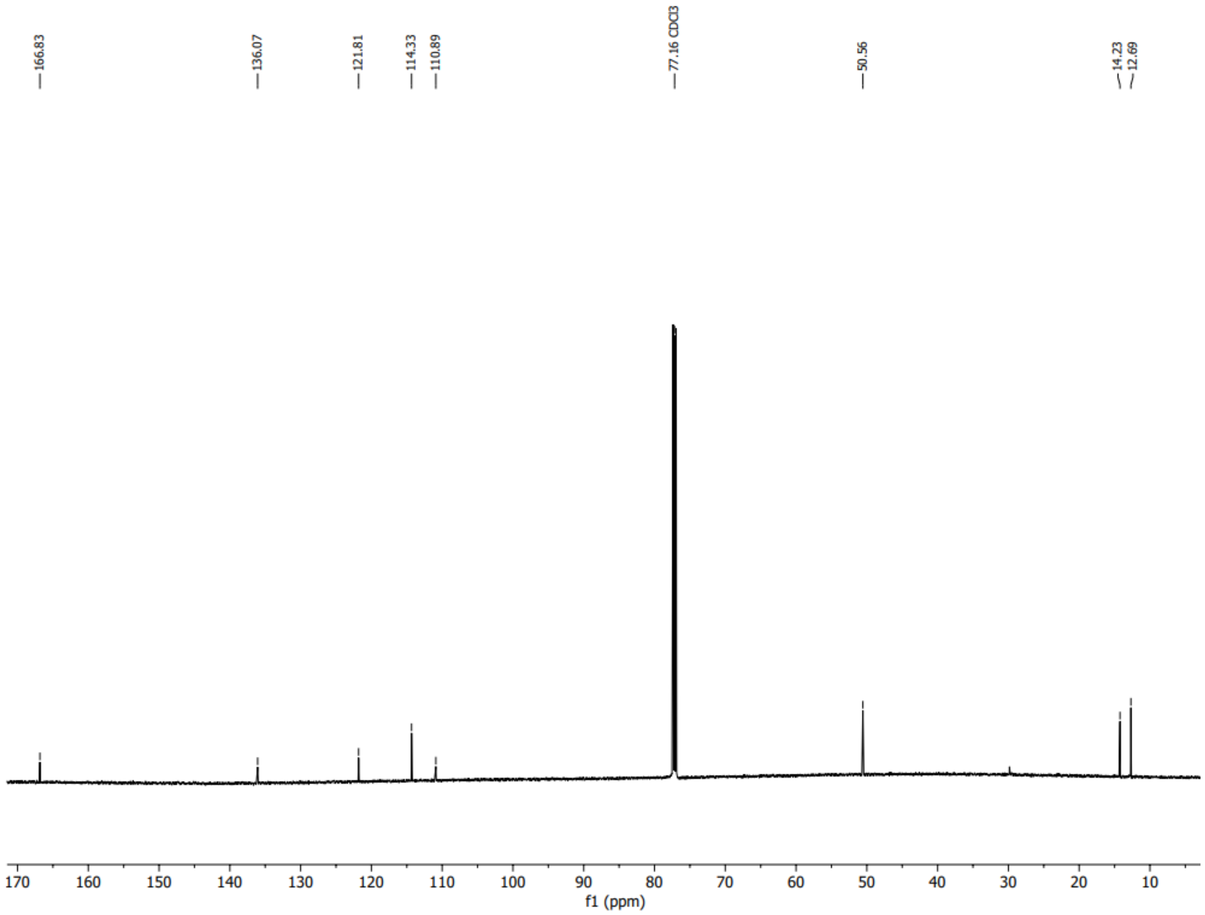

Figure S12B. Analysis of isolated pyrrole (1). 13C NMR (150 MHz, CDCl3): δC 166.8, 136.1, 121.8, 114.3, 110.9, 50.6, 14.2, 12.7.

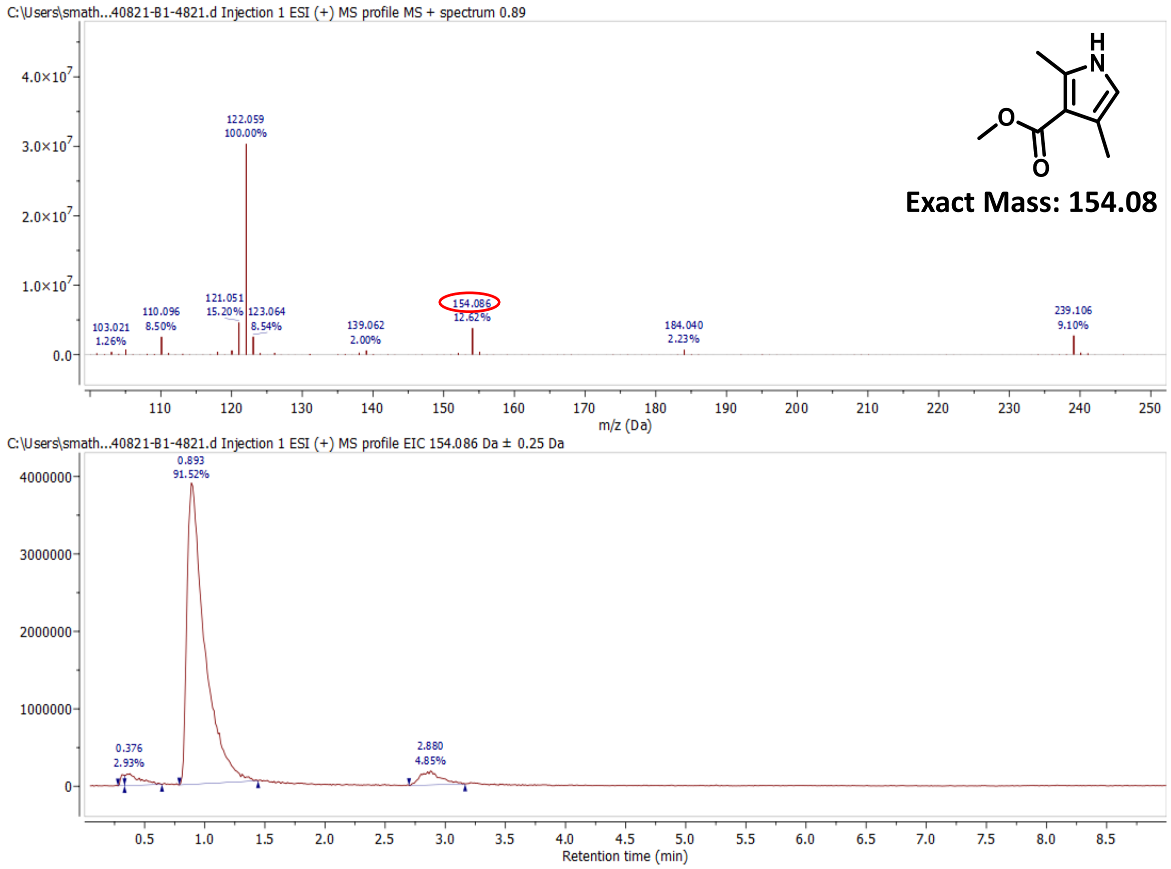

Figure S13. Analysis of isolated pyrrole (1). LC-TOF MS analysis of pyrrole using glycine (Top panel). Extracted ion chromatogram of pyrrole using glycine (Bottom panel).

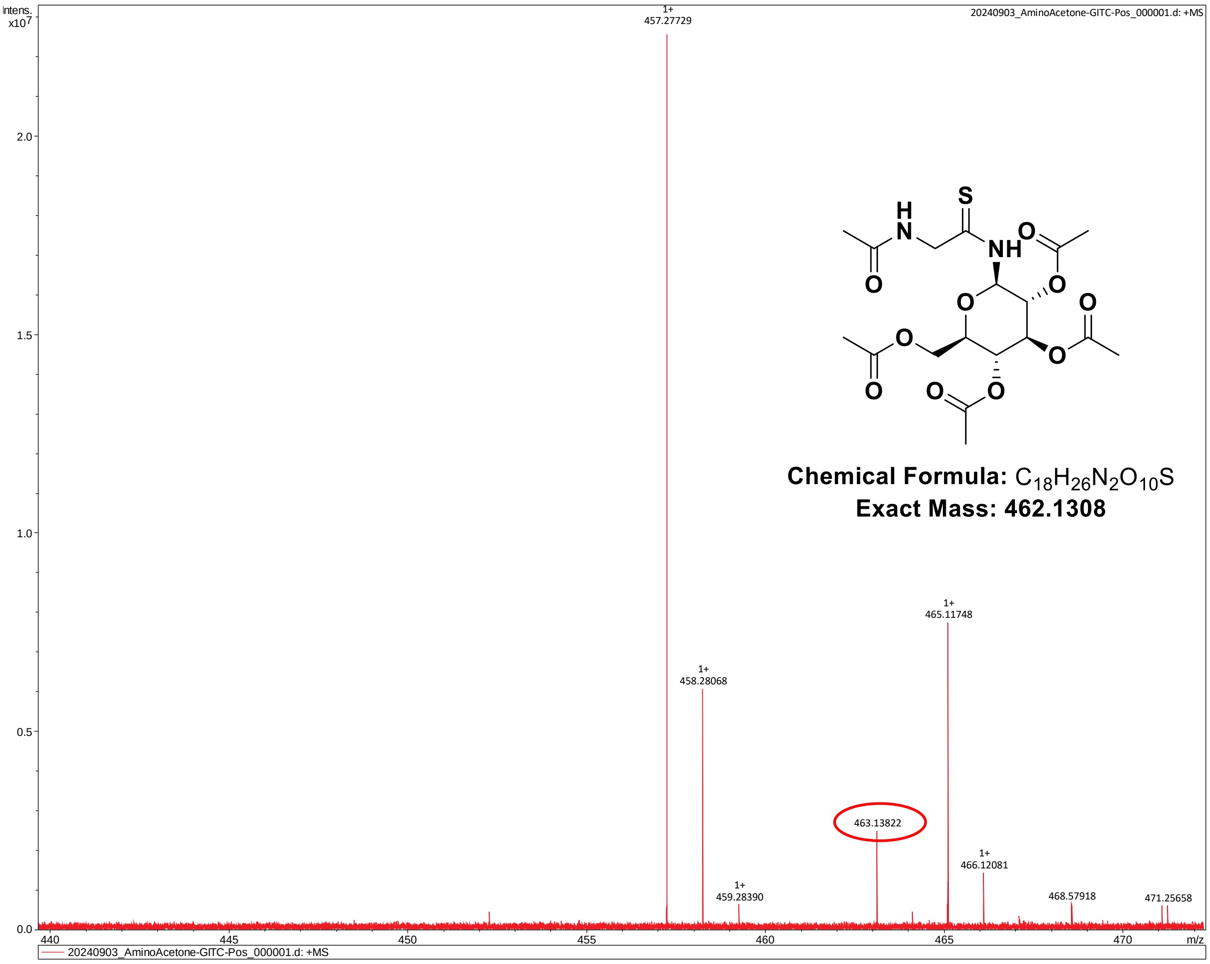

Figure S14. Mass spectrometry analysis of the ThAOS V79A variant incubated with Gly and acetyl-CoA. The glycine aminoacetone product derivatized using 2,3,4,6-tetra-O-acetyl-β-d-glucopyranosyl isothiocyanate (GITC). The derivatization of was performed by mixing the reaction mixture with GITC (1 mM) and incubated at 30 °C for 30 minutes. The ion with *m/z* = 463.13822 (circled) corresponds to the $[M+H]^+$ ion under electrospray conditions.

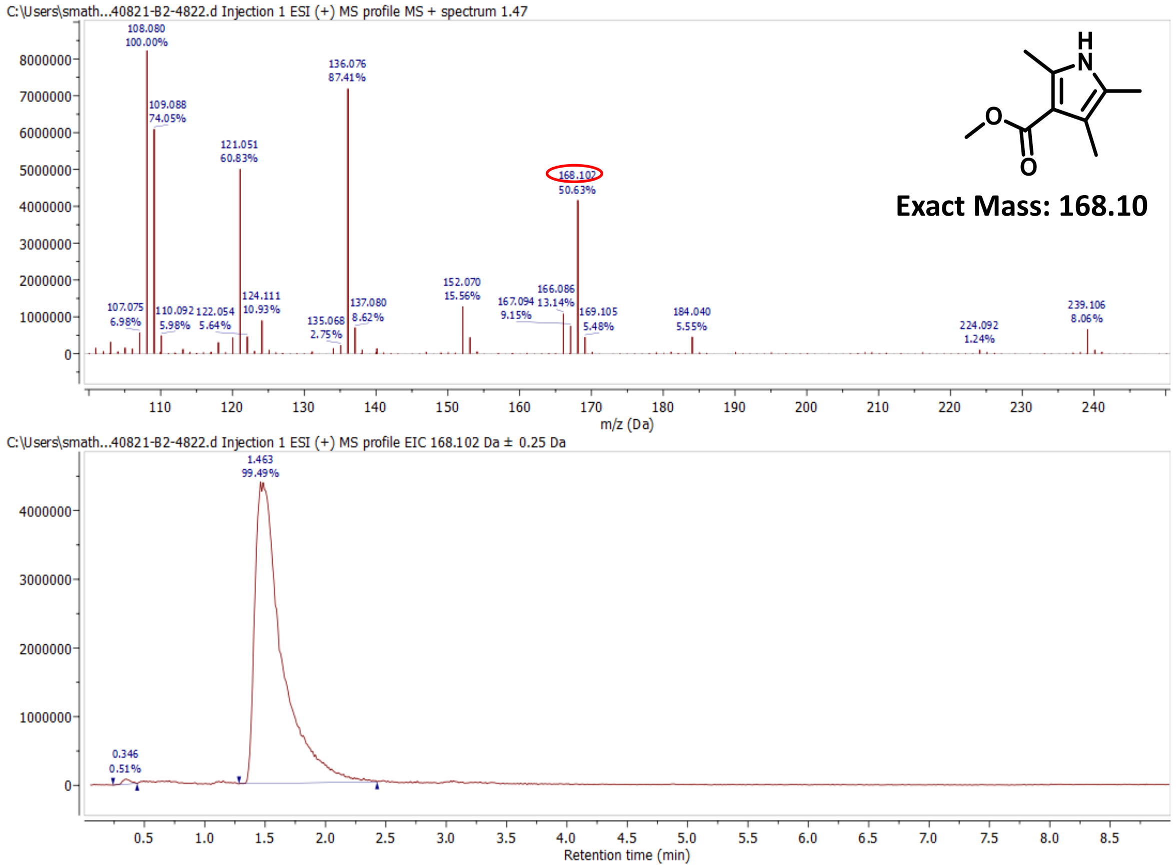

Figure S15. Formation of pyrrole from l-Alanine. LC-TOF MS analysis of pyrrole derived from L-Ala (Top panel). Extracted ion chromatogram (Bottom panel).

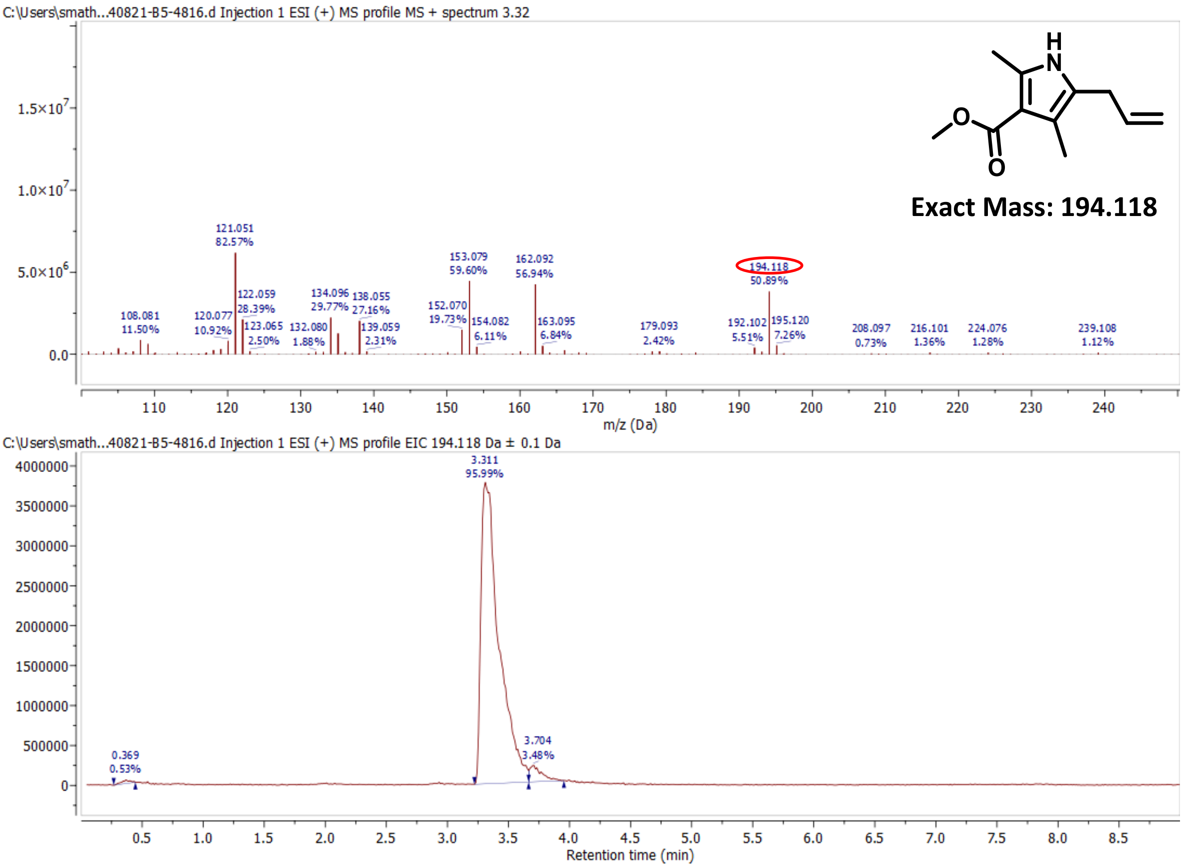

Figure S16. Formation of pyrrole from l-Allyglycine. LC-TOF MS analysis of pyrrole derived from L-Alg (Top panel). Extracted ion chromatogram (Bottom panel).

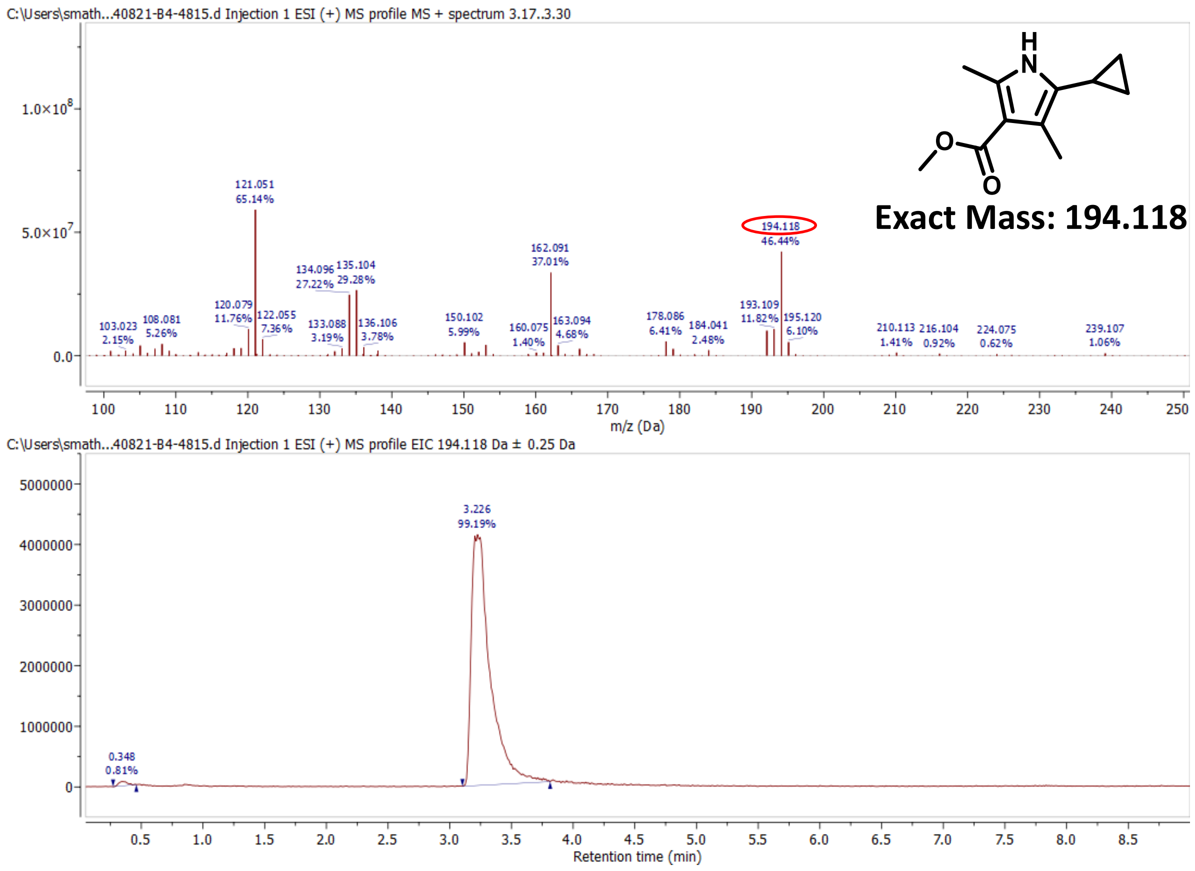

Figure S17. Formation of pyrrole from l-Cyclopropylglycine. LC-TOF MS analysis of pyrrole derived from L-Cpg (Top panel). Extracted ion chromatogram (Bottom panel).

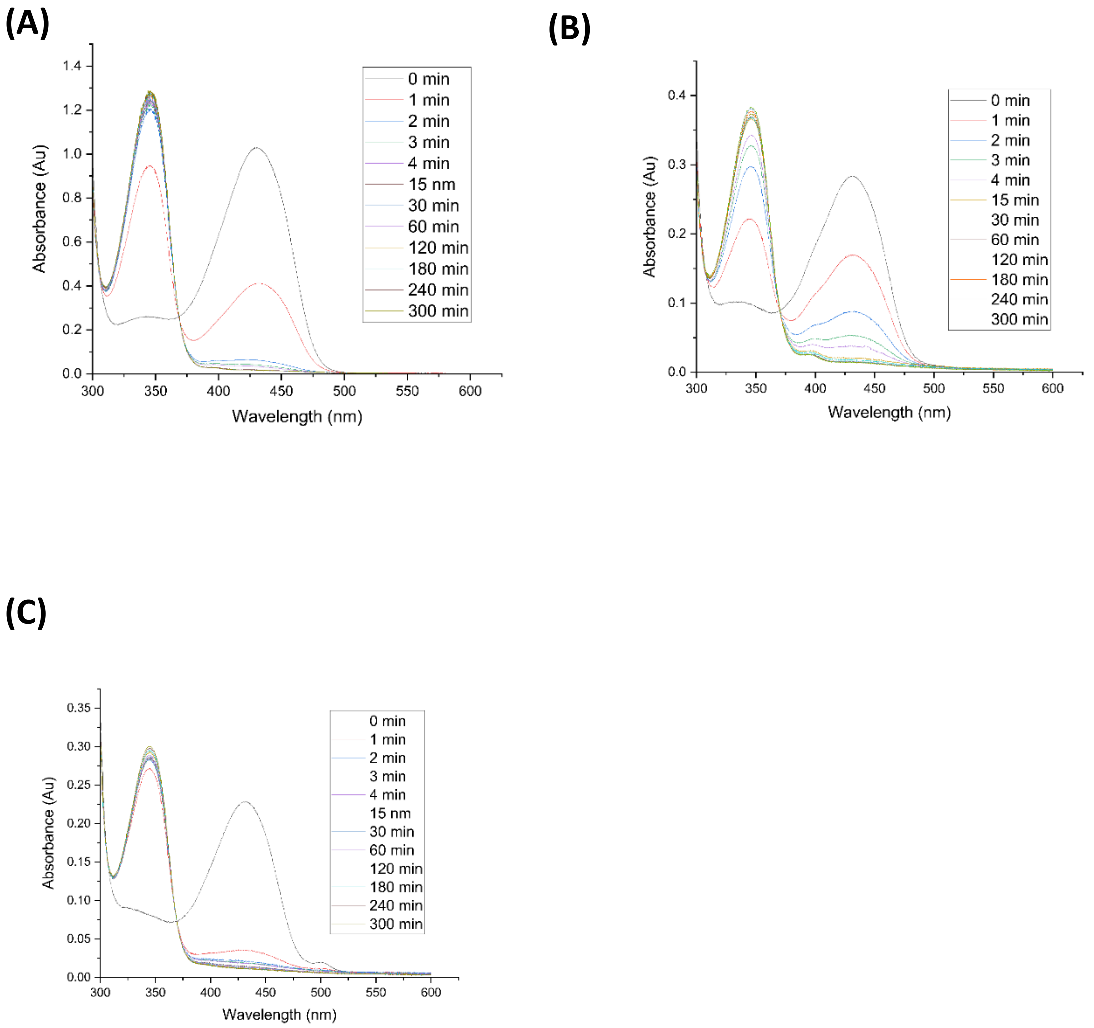

Figure S18. UV-vis analysis of L-Pen binding to ThAOS. The L-Pen was added to **(A)** wild type *Th*AOS **(B)** *Th*AOS V79A and **(C)** *Th*AOS V79G. The samples were incubated at room temperature and spectra recorded from 0-300 mins.

Structural studies of the *Th*AOS V79A variant, L-Pen ligand binding and comparison with similar enzymes.

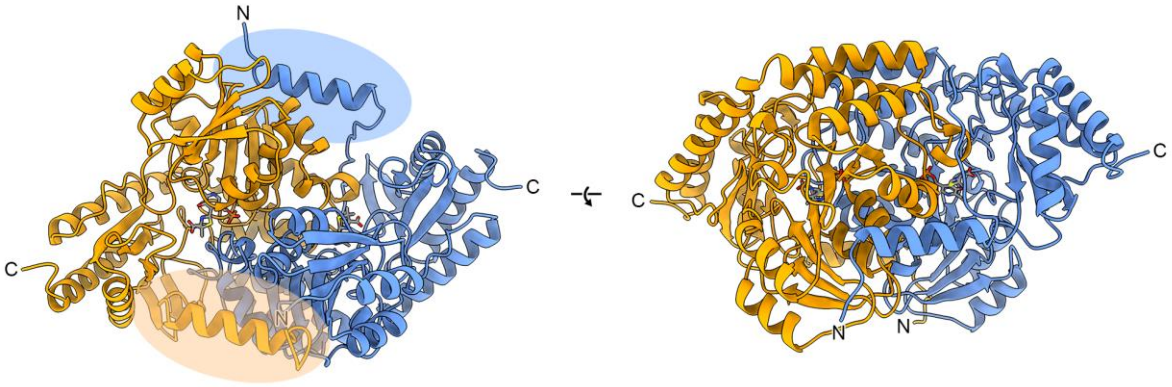

Figure S19A. Overall structure of ThAOS V79A. The crystallographic dimer of *Th*AOS V79A is shown in cartoon representation with the two chains coloured orange and blue. The thiazolidine ligand is shown in stick representation with grey carbon atoms. The extended N-terminal regions are highlighted with orange and blue ellipses. Figure created using ChimeraX version 1.6.1.

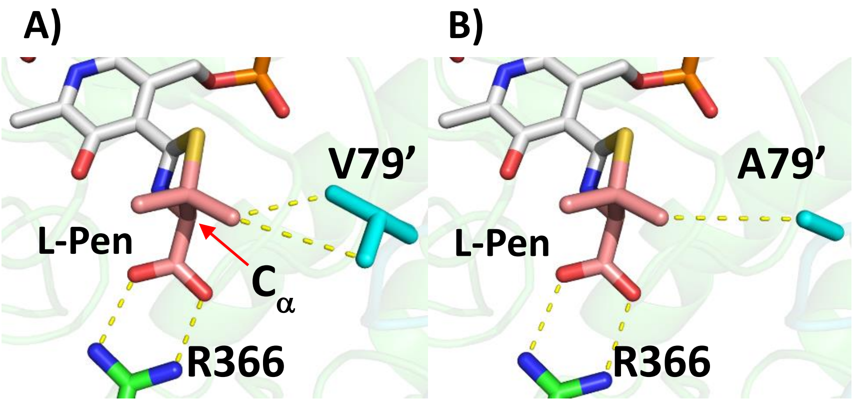

Figure S19B. Side-chain: L-Pen ligand interactions. Interaction of the ligand L-Pen with (A) Val79' and Arg366 in the overlaid scaffold structure of WT *Th*AOS and (B) Ala79' in the ligand-bound crystal structure of *Th*AOS V79A.

Comparison of penicillamine inhibitor binding in structural homologues.

Searching the PDB for the L- and D- forms of the penicillamine ligand identifies two unpublished structures of cysteine desulfurase (CSD) enzymes with structural homology to *Th*AOS with bound penicillamine ligand: NifS from *Helicobacter pylori* (PDB ID: 7XES); and SufS from *Bacillus subtilis* (PDD ID: 7XEN). There is also a structure of the SufS without penicillamine (PDB ID: 7XEN). The monomers of these two proteins align to *Th*AOS V79A with overall RMSD Cα of 1.3 Å (7XES) and 1.2 Å (7XEN) (Fig. S20). The primary structural difference between *Th*AOS and the CSD enzymes is in the position of the N-terminal region of these proteins and an extended beta-elbow that is present in the cysteine desulfurases (Fig. S20A). In *Th*AOS-V79A, the first 40 amino acids adopt an alpha helix and extended loop arrangement, forming a large interface with the partner chain (Fig. S20B); whereas, in the cysteine desulfurase enzymes the N-terminal region is shorter; and, in the case of the *B. subtilis* enzyme, it forms a twisted helix that mainly participates in interactions with its own chain (Fig. 20). These regions are shifted by a rotation of 110° between the cysteine desulfurase enzymes and *Th*AOS. The beta-elbow region in the cysteine desulfurase enzymes participates in the dimerisation interface and due to this additional region, the quaternary arrangement of these enzymes differs from *Th*AOS (Fig. 21A-B). It appears that it is the entrance to this cavity that has been engineered in the more active *Th*AOS V79 variants.

The active sites of NifS and SufS both have a bound L-pen ligand captured in the external aldimine form between the penicillamine and PLP cofactor (Fig. 21A). There position of the PLP and L-pen is well conserved between the three proteins other than the ring closure to form the thiazolidine in the *Th*AOS. There is a clear cavity at the dimer interface in each enzyme where the substrate can bind (Fig. 21B). The wider active site and ligand binding regions of the three proteins differ considerably, with distinct ligand binding tunnels in the three enzymes. There is only 20% sequence identity between *Th*AOS and the bacterial CSDs, and residue conservation in the active site is limited to histidine (H136), and arginine (R366); the active arginine (R243 in *Th*AOS) is conserved, but in different sequence positions in the NifS and SufS proteins (Fig. S22).

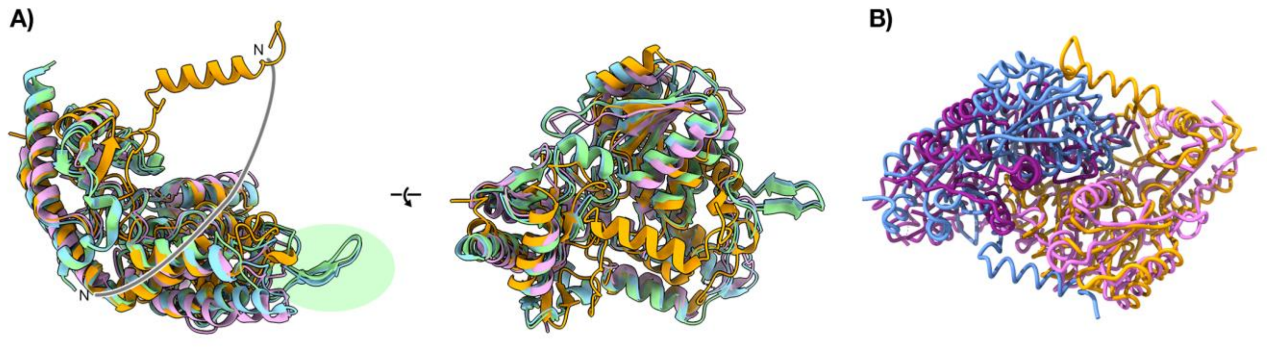

Figure S20. Structural alignment of ThAOS V79A and homologous cysteine desulfurase (CSD) enzymes (CSDs). **(A)** Secondary structure overlay of *Th*AOS-V79A (orange) and cysteine desulfurase (CSD) structural homologues from *H. pylori* (PDB ID: 7XES (pink)) and *B. subtilis* (PDB IDs: 7XEL (blue), 7XEN (green). The change in position of the N-terminal region is depicted with a grey curve). The extended beta-elbow present in the CSDs is highlighted with a green ellipse. **(B)** Dimer view showing position of the N-terminal helices in relation to partner chains. The *Th*AOS shown in blue/orange and *H. pylori* CSD in purple/pink. Figure created using ChimeraX version 1.6.1.

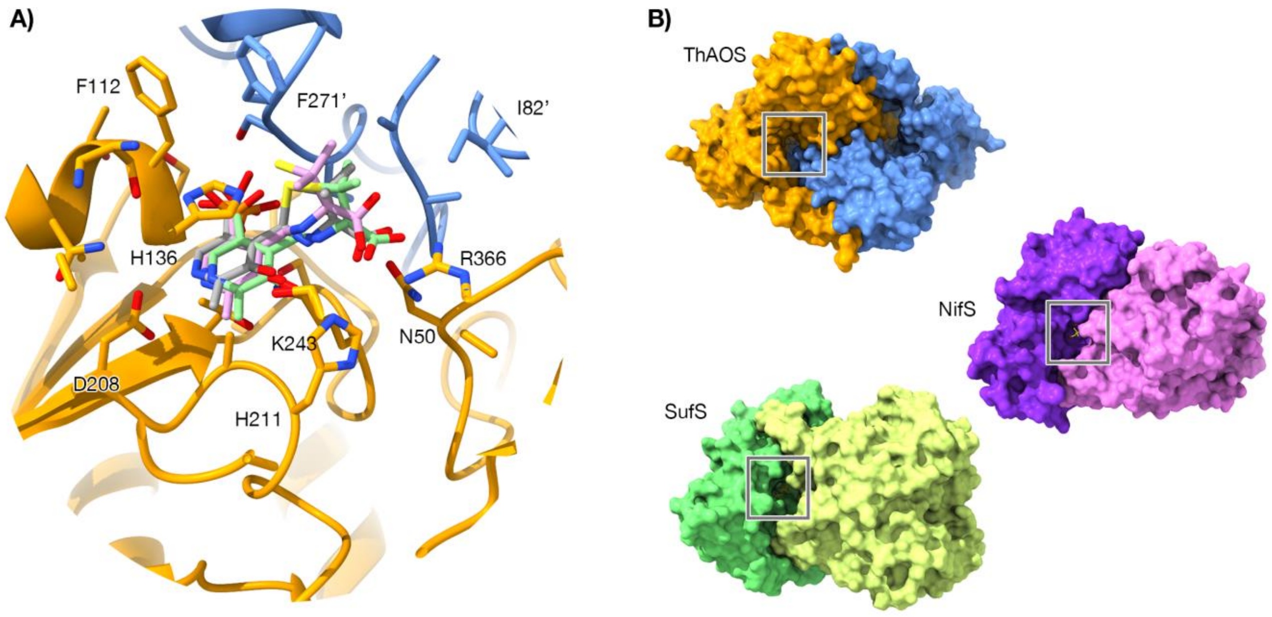

Figure S21. Binding of L*-Pen in ThAOS V79A and cysteine desulfurases (CSDs).* **(A)** The position of the PLP-penicillamine external aldimine from the NifS and SufS proteins are depicted in green and pink sticks respectively. **(B)** Surface view of *Th*AOS, SufS and NifS, showing the position of active site cleft in relation to dimer interface (grey box).

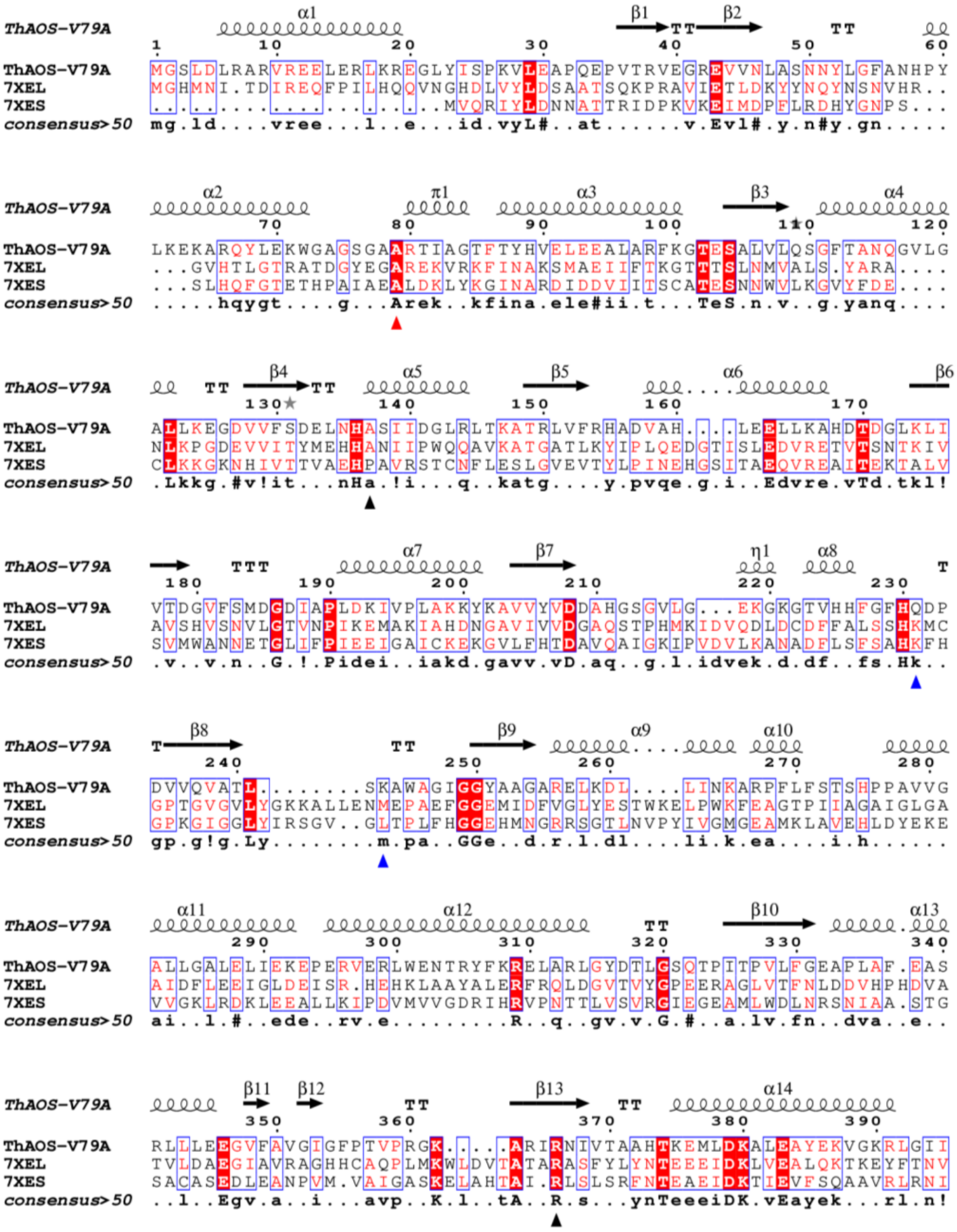

Figure S22. Multiple sequence alignment of ThAOS V79A variant and cysteine desulfurase (CysD) enzymes. Multiple sequence alignment produced using Multalin and rendered in ESPript. Secondary structure elements for *Th*AOS V79A are depicted above the alignment. Conserved PLP and substrate binding residues are highlighted with black arrows, the PLP-binding lysine residues are highlighted with blue arrows, and the *Th*AOS V79A variant site with a red arrow.

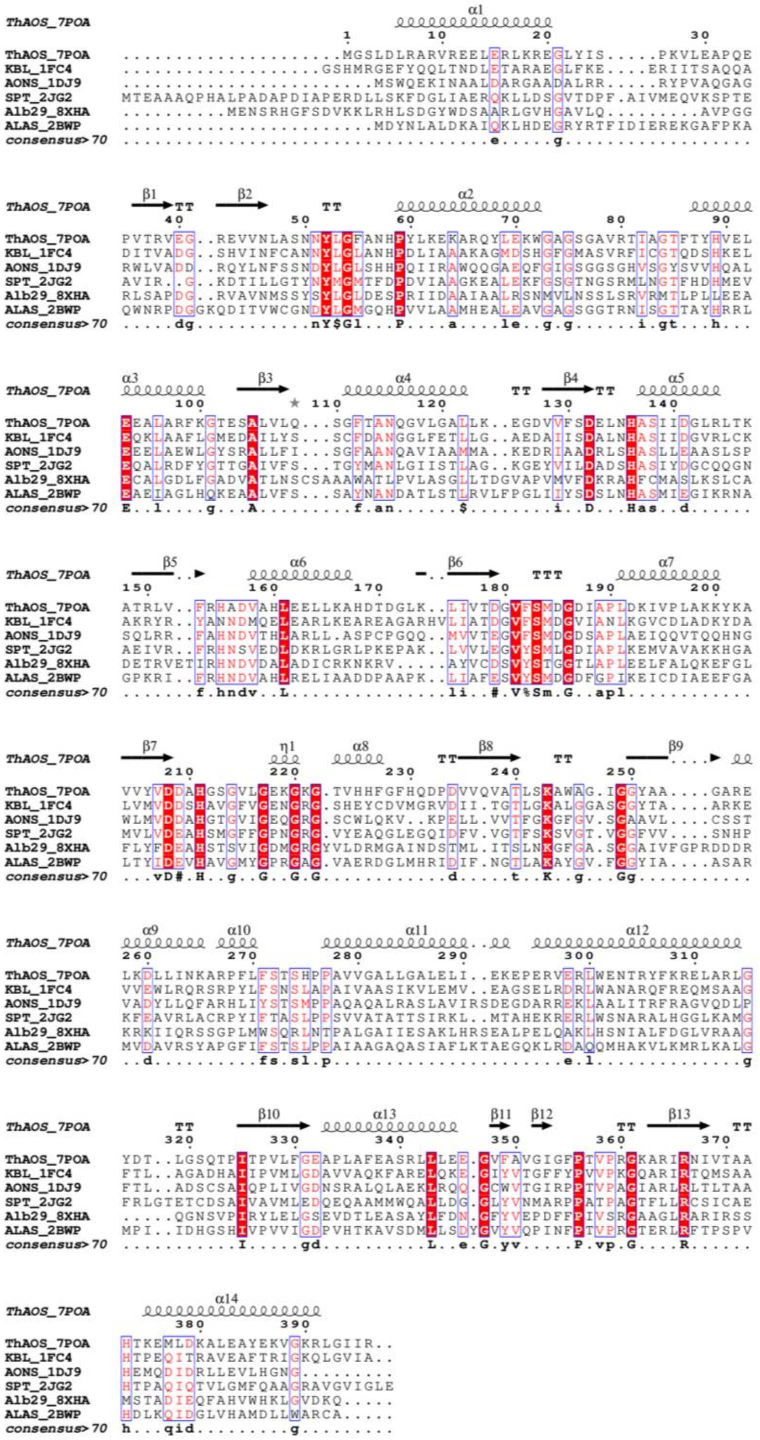

Figure S23. Multiple sequence and structural alignment of various AOS biocatalysts. This highlights the residues equivalent to position V79 of *Th*AOS. The KBL enzyme from *E. coli* (1FC4) retains the valine residue whereas the AONS enzyme from *E. coli* and SPT enzyme from *S. paucimobilis* (PDB: 2JG2) have a serine side chain. An Alb29 enzyme from *S. albogriseolus* (8XHA) has a leucine and the ALAS enzyme from *R. capsulatus* has a threonine at this equivalent position (PDB: 2BWP).

Tables S1-S6

Primer	Sequence
V79A F	5’-TAGCGGTGCG**C**GGCGTACCATCGCGGGCAC-3'
V79A R	5’-CGCGATGGTACGCC**G**CGCACCGCTACCCGC-3'
V79G F	5’- TAGCGGTGCG**G**GGCGTACCATCGCGGGCAC -3'
V79G R	5’- CGCGATGGTACGCC**C**CGCACCGCTACCCGC -3'
V79X F 1	5’-TAGCGGTGCG**NDT**CGTACCATCGCGGGCAC-3’
V79X R 1	5’-CGCGATGGTACG**AHN**CGCACCGCTACCCGC-3’
V79X F 2	5’-TAGCGGTGCG**VHG**CGTACCATCGCGGGCAC-3’
V79X R 2	5’-CGCGATGGTACG**CDB**CGCACCGCTACCCGC-3’
V79W F	5’-TAGCGGTGCG**TGG**CGTACCATCGCGGGCAC-3’
V79W R	5’-CGCGATGGTACG**CCA**CGCACCGCTACCCGC-3’
V79D F	5’-TAGCGGTGCG**GAT**CGTACCATCGCGGGCAC-3’
V79D R	5’-CGCGATGGTACG**ATC**CGCACCGCTACCCGC-3’
V79E F	5’-TAGCGGTGCG**GAA**CGTACCATCGCGGGCAC-3’
V79E R	5’-CGCGATGGTACG**TTC**CGCACCGCTACCCGC-3’
V79CFSY F	5’-TAGCGGTGCG**TNC**CGTACCATCGCGGGCAC-3’
V79CFSY R	5’-CGCGATGGTACG**GNA**CGCACCGCTACCCGC-3’
V79KQ F	5’-TAGCGGTGCG**MAA**CGTACCATCGCGGGCAC-3’
V79KQ R	5’-CGCGATGGTACG**TTK**CGCACCGCTACCCGC-3’
V79T F	5’-TAGCGGTGCG**ACA**CGTACCATCGCGGGCAC-3’
V79T R	5’-CGCGATGGTACG**TGT**CGCACCGCTACCCGC-3’
V79IL F	5’-TAGCGGTGCG**MTC**CGTACCATCGCGGGCAC-3’
V79IL R	5’-CGCGATGGTACG**GAK**CGCACCGCTACCCGC-3’
V79H F	5’-TAGCGGTGCG**CAT**CGTACCATCGCGGGCAC-3’
V79H R	5’-CGCGATGGTACG**TAG**CGCACCGCTACCCGC-3’
V79R F	5’-TAGCGGTGCG**CGT**CGTACCATCGCGGGCAC-3’
V79R R	5’-CGCGATGGTACG**ACG**CGCACCGCTACCCGC-3’
A78A V79V I82A F	5’-TGC**A**GT**T**CGTACC**GCT**GCGGGCACCTTCACCTATC-3'
A78A V79V I82A R	5’-**AGC**GGTACG**A**AC**T**GCACCGCTACCCGCACC-3'
V79A T80T I82A F	5’-TGCGGGTCGTAC**TGCD**GCGGGCACCTTCACCTATC-3’
V79A T80T I82A R	5’-**HGCA**GTACGACCCGCACCGCTACCCGCACC-3’
V79G T80T I82A F	5’-TGCGGGTCGTAC**TGCT**GCGGGCACCTTCACCTATC-3’
V79G T80T I82A R	5’-**AGCA**GTACGACCCGCACCGCTACCCGCACC-3’
S182S M183A F	5’-TTTAG**TGCT**GACGGTGATATCGCGCCGCTGG-3’
S182S M183A R	5’-TATCACCGTC**AGCA**CTAAAAACGCCGTCGGTCAC-3’
F280A F	5’-GGTGCT**TGC**CAGGAACGGACGCGCTTTGTTAATC-3’
F280A R	5’-CGTTCCTG**GCA**AGCACCAGCCACCCGCCGG-3’

Table S1. Primer sequences used to generate all mutants used in this study. Mutated bases are highlighted in bold.

*Th*AOS	Substrate	k_{cat} (s^{-1})	K_M (mM)	k_{cat}/K_M ($s^{-1}mM^{-1}$)	× fold improved
**WT**	L-Aba	0.06 ± 0.01	24.2 ± 5.32	2.48×10^{-2}	-
**WT**	L-Ala	0.12 ± 0.01	1.63 ± 0.64	6.33×10^{-2}	-
**WT**	Gly	0.49 ± 0.03	2.47 ± 0.45	1.97×10^{-1}	-
**WT**	L-Ser	0.11 ± 0.01	4.94 ± 0.85	2.33×10^{-2}	-
**V79A**	L-Aba	2.25 ± 0.08	3.12 ± 0.35	7.23×10^{-1}	29.2
**V79A**	L-Ala	1.23 ± 0.05	1.59 ± 0.25	7.73×10^{-1}	12.2
**V79A**	L-Asp	0.10 ± 0.01	51.6 ± 6.31	2.03×10^{-3}	-
**V79A**	L-Cpg	0.17 ± 0.02	11.3 ± 2.86	1.54×10^{-2}	-
**V79A**	Gly	3.89 ± 0.32	8.36 ± 1.73	4.65×10^{-1}	2.4
**V79A**	L-Hsr	0.44 ± 0.03	2.90 ± 0.57	1.53×10^{-1}	-
**V79A**	L-Ile	0.17 ± 0.01	33.4 ± 4.48	5.01×10^{-3}	-
**V79A**	L-Pra	0.25 ± 0.01	8.92 ± 0.69	2.77×10^{-2}	-
**V79A**	L-Ser	3.81 ± 0.13	2.52 ± 0.30	1.51×10^{0}	64.8
**V79A**	L-Thr	0.09 ± 0.00	2.27 ± 0.14	4.13×10^{-2}	-
**V79A**	L-Val	0.21 ± 0.01	33.4 ± 0.01	6.23×10^{-3}	-
**V79G**	L-Aba	1.09 ± 0.04	0.83 ± 0.15	1.33×10^{0}	53.6
**V79G**	L-Ala	2.39 ± 0.15	2.46 ± 0.05	9.72×10^{-1}	15.4
**V79G**	L-Asp	0.11 ± 0.01	22.5 ± 3.76	4.84×10^{-3}	-
**V79G**	L-Cpg	0.74 ± 0.04	4.18 ± 0.71	1.77×10^{-1}	-
**V79G**	Gly	5.12 ± 0.43	9.52 ± 1.91	5.38×10^{-1}	2.7
**V79G**	L-Hsr	0.72 ± 0.04	3.08 ± 0.62	2.34×10^{-1}	-
**V79G**	L-Ile	0.12 ± 0.01	17.3 ± 3.91	7.03×10^{-3}	-
**V79G**	L-Pra	0.07 ± 0.00	0.78 ± 0.15	8.99×10^{-2}	-
**V79G**	L-Ser	5.74 ± 0.43	4.28 ± 0.96	1.34×10^{0}	57.5
**V79G**	L-Thr	0.23 ± 0.01	2.58 ± 0.25	9.37×10^{-2}	-
**V79G**	L-Val	0.35 ± 0.01	2.62 ± 0.29	1.32×10^{-1}	-

Table S2. Full kinetic characterisation of ThAOS V79A and ThAOS V79G with various amino-acid substrates. The assay was carried out using acetyl-CoA (1 mM) and monitored with the DTNB 412 nm assay at 50 °C. Michaelis-Menten fitting was performed in OriginLab 2019. "× improvement" is calculated as the improvement in catalytic efficiency k_{cat}/K_M relative to the preceding mutant.

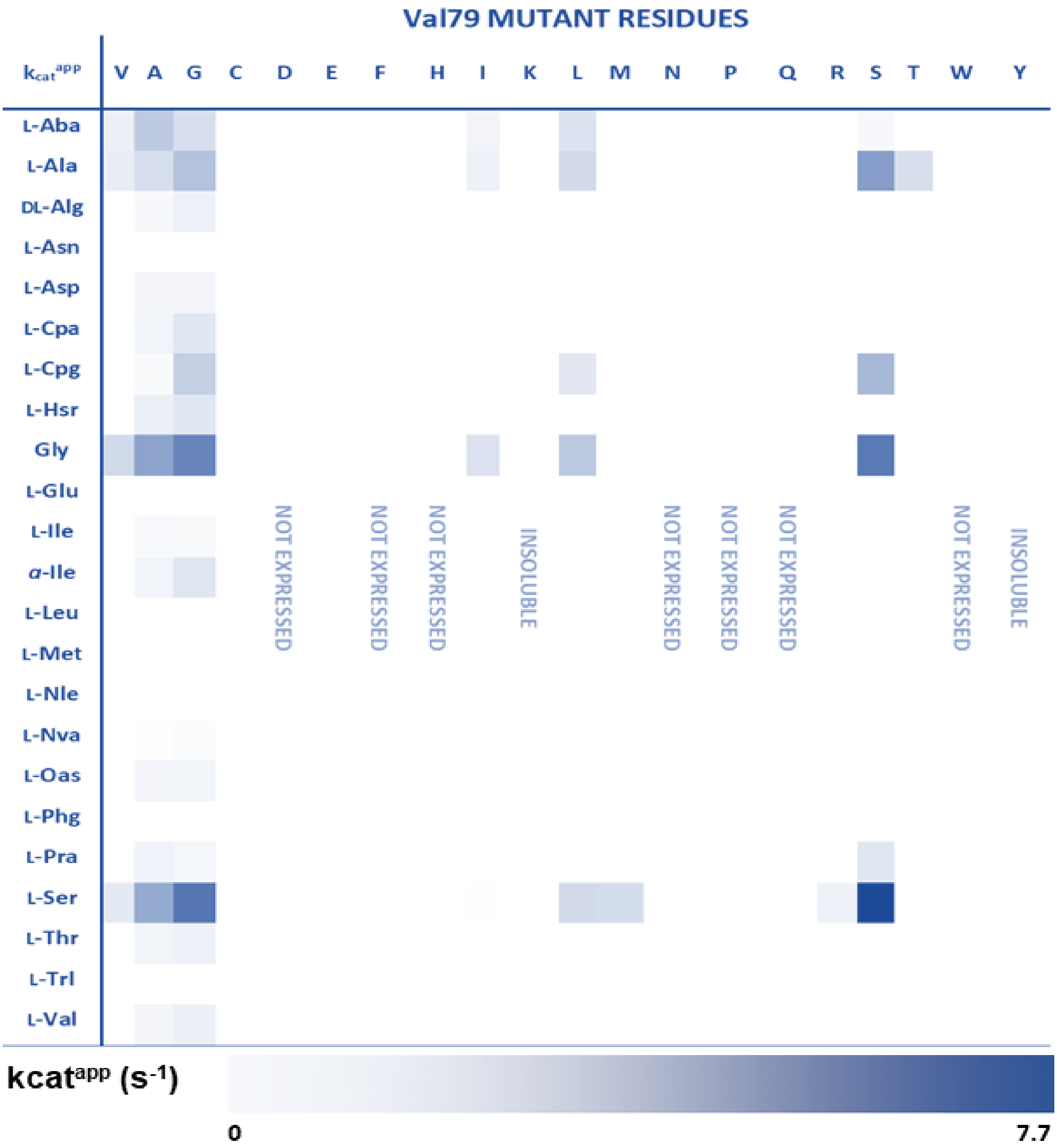

Table S3. Summary of saturation mutagenesis at ThAOS V79. Comment on expression profile and whether variant was soluble/insoluble. Heat-map of AOS condensation activity with acetyl-CoA by *Th*AOS V79X mutants, as determined by the DTNB activity assay with 16 mM and 1 mM acetyl-CoA at 50 °C. The scale bar and heat map describe the activity, the darkest blue in this figure corresponds to a k_{cat}^{app} of 7.70 s^{-1} and white corresponds to an inactive substrate pairing.

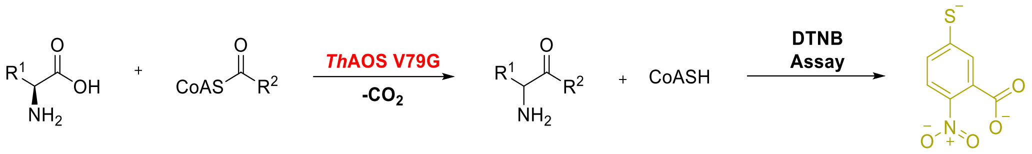

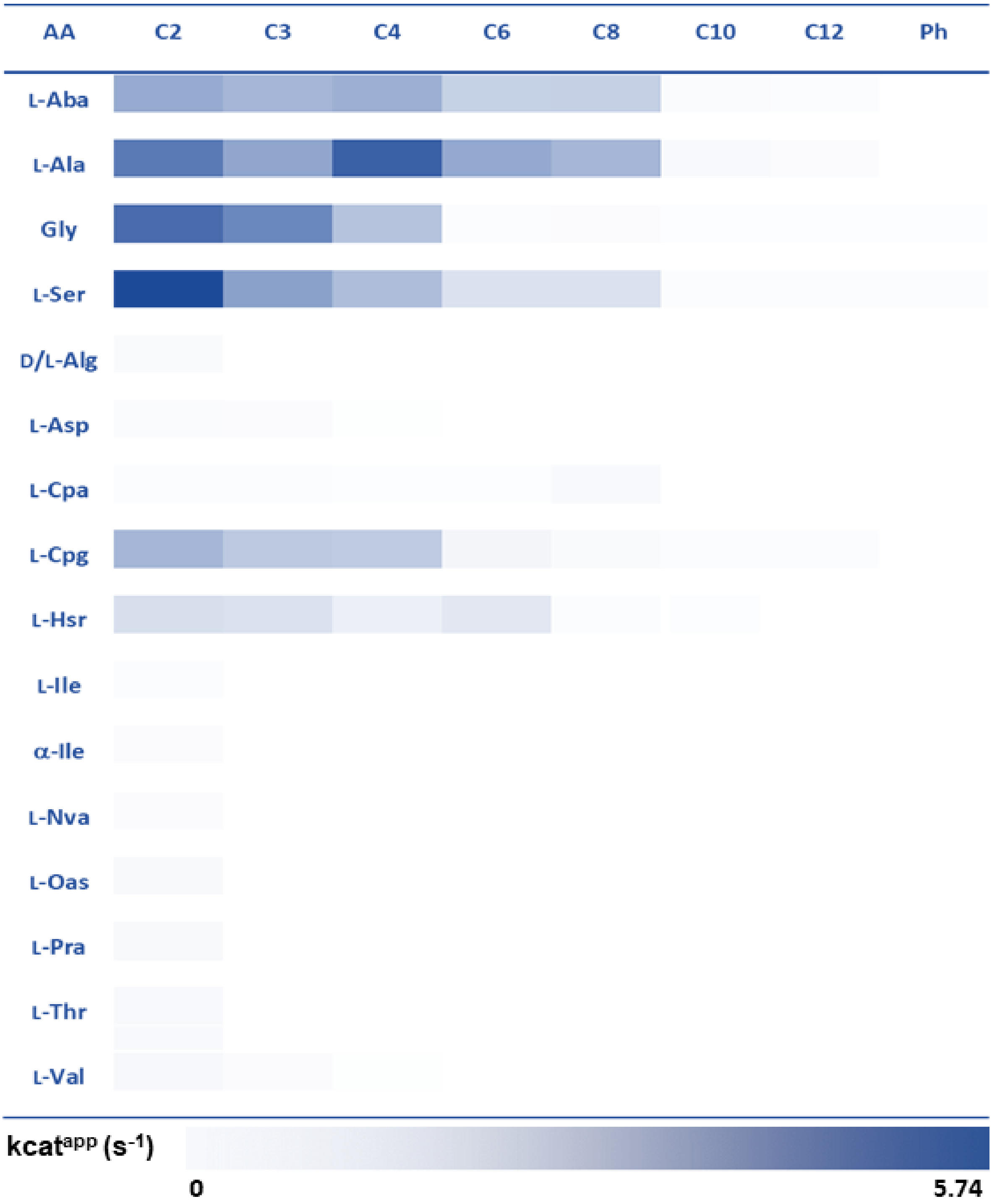

Table S4. Heat map illustrating the full Claisen-condensation substrate range of ThAOS V79G. Activity was determined in the same way as detailed in Table 1. The scale bar and heat map describe the activity, the darkest blue corresponds to a specific turnover number of 5.74 s^{-1} and white denotes an inactive substrate pair.

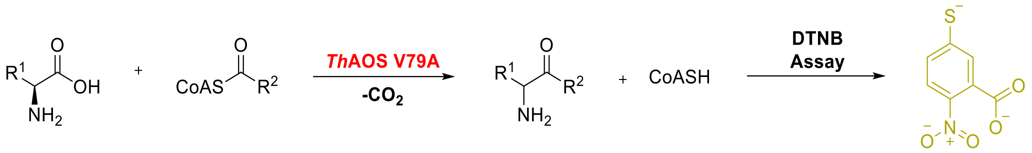

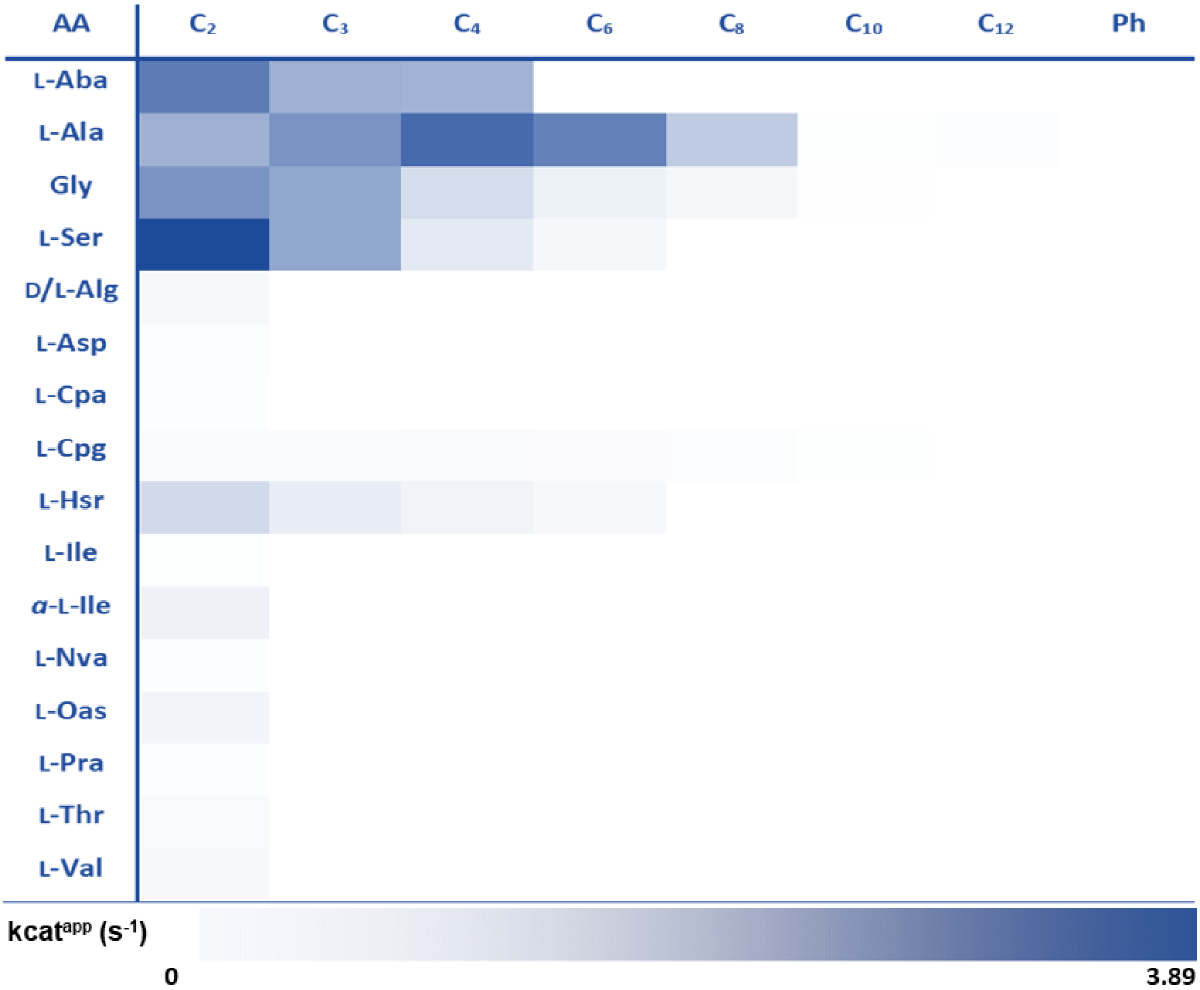

Table S5. Heat-map of the activity of ThAOS V79A with a panel of L-amino-acid/acyl-CoA substrate pairings. The scale bar and heat map describe the activity, the darkest blue in this heatmap (L-Ser with acetyl-CoA) corresponds to a k_{cat}^{app} of 3.89 s^{-1}, and white denotes an inactive substrate.

X-ray data collection and processing statistics*	
	*Th*AOS-V79A
Beamline	I04
Date	07/07/21
Wavelength (Å)	0.9794
Resolution (Å)	34.77 - 1.5 (1.54 - 1.5)
Space group	P1
**Unit cell parameters**	
a (Å)	57.43
b (Å)	63.94
c (Å)	65.97
α (°)	64.80
β (°)	70.29
γ (°)	68.18
Unit-cell volume ($Å^3$)	198,813.84
Solvent content (%)	42.5
**Data scaling and merging**	
No. of measured reflections	417,255 (39,811)
No. of independent reflections	118,545 (11,721)
Completeness (%)	96.06 (94.51)
Redundancy	3.5 (3.4)
$CC_{1/2}$	1 (0.542)
<I>/<σ(I)>	16.73 (1.46)
R_{merge}	0.029 (0.82)
R_{meas}	0.034 (0.98)
R_{pim}	0.018 (0.53)
**Model refinement statistics**	
Reflections in refinement	118,545
Reflections in free set	5892
Rwork (%)	15.9 (28.2)
Rfree# (%)	19.2 (29.6)
Clashscore	4.59
Rotamer outliers (%)	2.2
No. of non-H atoms	6,812
No. of protein, atoms	6,161
No. of solvent atoms	555
No. of ligand atoms	96
**RMS deviation from ideal values**	
Bond angles (°)	0.0149
Bond lengths (Å)	2.0690
**Average B factors ($Å^2$)**	
Protein	26.55
Solvent	33.98
Ligand	33.90
**Ramachandran plot**	
Most favoured regions (%)	97.7
Allowed regions (%)	2.3
PDB ID	8S1Y

*(Values in parenthesis are for the highest resolution shell).

#5% of the randomly selected reflections excluded from refinement.

+Calculated using MOLPROBITY

Table S6. Crystallographic data collection and refinement statistics.

References

1. Liu, H. & Naismith, J. H. An efficient one-step site-directed deletion, insertion, single and multiple-site plasmid mutagenesis protocol. *BMC Biotechnol* **8**, 91 (2008).
2. Kille, S. *et al.* Reducing codon redundancy and screening effort of combinatorial protein libraries created by saturation mutagenesis. *ACS Synth Biol* **2**, 83-92 (2013).
