# Supplementary material for: Rational engineering of a thermostable α-oxoamine synthase biocatalyst expands the substrate scope and synthetic applicability
Source: Commun Chem. 2025 Mar 13;8:78. doi: 10.1038/s42004-025-01448-8 (PMC11906848; doi:10.1038/s42004-025-01448-8)
Supplement: Supplementary file 3 — Description of Additional Supplementary Files [file 42004_2025_1448_MOESM3_ESM.pdf]

Description of Additional Supplementary Files

File name: Supplementary Data 1

Description: Dataset for Table 1

File name: Supplementary Data 2

Description: Dataset for Table 2
